# Neutrophil extracellular traps in sheep mastitis

**DOI:** 10.1186/s13567-015-0196-x

**Published:** 2015-06-18

**Authors:** Salvatore Pisanu, Tiziana Cubeddu, Daniela Pagnozzi, Stefano Rocca, Carla Cacciotto, Alberto Alberti, Gavino Marogna, Sergio Uzzau, Maria Filippa Addis

**Affiliations:** Porto Conte Ricerche, Alghero, (SS) Italy; Dipartimento di Medicina Veterinaria, Università di Sassari, Sassari, Italy; Istituto Zooprofilattico Sperimentale G, Pegreffi Sassari, Italy

## Abstract

Neutrophil extracellular traps (NETs) are structures composed of DNA, histones, and antimicrobial proteins that are released extracellularly by neutrophils and other immune cells as a means for trapping and killing invading pathogens. Here, we describe NET formation in milk and in mammary alveoli of mastitic sheep, and provide a dataset of proteins found in association to these structures. Nucleic acid staining, immunomicroscopy and fluorescent in-situ hybridization of mastitic mammary tissue from sheep infected with *Streptococcus uberis* demonstrated the presence of extranuclear DNA colocalizing with antimicrobial proteins, histones, and bacteria. Then, proteomic analysis by LTQ-Orbitrap Velos mass spectrometry provided detailed information on protein abundance changes occurring in milk upon infection. As a result, 1095 unique proteins were identified, of which 287 being significantly more abundant in mastitic milk. Upon protein ontology classification, the most represented localization classes for upregulated proteins were the cytoplasmic granule, the nucleus, and the mitochondrion, while function classes were mostly related to immune defence and inflammation pathways. All known NET markers were massively increased, including histones, granule proteases, and antimicrobial proteins. Of note was the detection of protein arginine deiminases (PAD3 and PAD4). These enzymes are responsible for citrullination, the post-translational modification that is known to trigger NET formation by inducing chromatin decondensation and extracellular release of NETs. As a further observation, citrullinated residues were detected by tandem mass spectrometry in histones of samples from mastitic animals. In conclusion, this work provides novel microscopic and proteomic information on NETs formed in vivo in the mammary gland, and reports the most complete database of proteins increased in milk upon bacterial mastitis.

**Electronic supplementary material:**

The online version of this article (doi:10.1186/s13567-015-0196-x) contains supplementary material, which is available to authorized users.

## Introduction

Mastitis is an inflammation of the mammary gland typically consequent to bacterial infection. In dairy animals, it is generally accompanied by reduced milk quantity and quality and by an increase in the number of cells in milk, defined as the somatic cell count (SCC). In sheep, mastitis is mainly due to infection by gram-positive pathogens, including staphylococci, streptococci, and enterococci [1-3]. Gram-negative pathogens, mainly enterobacteriaceae, can also cause sheep mastitis, although with significantly lower occurrences than in bovines [1,2]. Other relevant causal agents of mastitis in sheep are mycoplasmas, but since these etiologic agents do also cause other severe symptoms, including lameness, keratoconjunctivitis, and respiratory problems, some authors fail to consider them as mastitis agents. Nevertheless, mycoplasma infections, together with their consistent economical impact due to animal mortality and culling, reduce milk production, induce an increase in SCCs, and cause deterioration of milk quality [4].

Somatic cells are typically represented by epithelial cells, neutrophils, macrophages, and lymphocytes as the major cell types [5]. Their physiological levels in sheep milk are still the subject of controversies; in fact, issues remain on the fluctuations of this parameter due to numerous factors other than mastitis, such as management practices, stage of lactation, parity, and presence of lentiviral infections, to name a few [6,7]. Therefore, their physiological numbers and cell type patterns, as well as the SCC threshold to be considered for diagnostic purposes, are not yet well defined in this ruminant species. Typically, however, in milk of healthy sheep at the peak of lactation, epithelial cells and their fragments are the main cell type found; when infection or inflammation occur, a high number of neutrophils and macrophages are recruited into the alveolus lumen, causing a shift in the predominant cell type and a significant increase in the total SCC [5,8]. From studies in bovines and on the murine experimental model, it is known that recruitment of immune cells is triggered by entry of bacteria into the lumen and by recognition of pathogen-associated microbial patterns (PAMPs) by mammary epithelial cells (MECs) and alveolar macrophages [9-12]. This causes the release of chemotactic and antimicrobial agents from these cells, leading to the massive influx of neutrophils in milk. Notably, however, despite their key role in controlling infections in the mammary gland, studies carried out on bovine neutrophils isolated from milk have demonstrated reduced antimicrobial capabilities, likely due to an inhibitory effect exerted by this fluid on the phagocytic activity and on the generation of reactive oxygen species (ROS) [13,14]. Nevertheless, there is an alternative method by which neutrophils, but also other phagocytes and epithelial cells, can disarm and kill pathogens extracellularly: the release of extracellular traps (ETs) [12,15,16]. Neutrophil extracellular traps (NETs) are represented by a mesh of DNA, histones, antimicrobial proteins and proteinases, that entrap and inactivate the invading microorganisms without requiring a direct contact or an engulfment by the host cell [17,18]. In vitro studies have demonstrated that one of the key biochemical events in NET formation is the deimination of arginine residues in histones to citrullines, catalyzed by protein-arginine deiminase (PAD) [19]. This post-translational modification triggers decondensation of the associated chromatin that, together with rupture of the nuclear membrane and dissolution of the cytoplasmic granules, enables mixing of the NET components in the cytoplasm and their subsequent release, in a series of event defined as NETosis [20]. There is intense debate on the formation and role of NETs in innate host defence, as well as uncertainty about the fact that this phenomenon might occur as an active and tightly orchestrated host immune response or as a passive process, as well as if neutrophil death is a prerequisite for NET formation. Actually, it is also not completely clear whether nucleus integrity is compromised by the release of NETs, since mechanisms alternative to nuclear lysis have been proposed [21]. These uncertainties are also amplified by the fact that most studies have been carried out in vitro, due the difficulty to image NET formation while occurring in vivo [18].

In 2006, Lippolis et al. demonstrated that bovine neutrophils are able to release NETs in vitro, and that their formation is not inhibited by milk [22]. The same authors postulated that, since milk fat and proteins inhibit the key antibacterial functions of the neutrophil (that is, phagocytosis and oxidative burst), NET formation might indeed play a central role in mammary gland health [22]. Later on, the same research group reported that NET proteins are present in mastitic bovine milk in association with the milk fat globule (MFG) fraction [23]. In our recent proteomic studies on sheep mastitis, we detected in the MFG fraction several proteins that can be associated to presence of NETs, in light of the recent studies on the subject [24,25]. This suggests that further studies carried out on mastitic sheep tissues and milk might have the potential to provide useful and novel information on the in vivo formation and presence of NETs, by investigating the events occurring in the mammary alveolus during bacterial infection.

With this purpose, tissues and milk collected from sheep experimentally infected with *S. uberis* were subjected to DNA staining, immunomicroscopy, and in-situ hybridization experiments, highlighting the presence of extracellular DNA in association with antimicrobial proteins, histones, and bacteria. Then, an in-depth characterization of the milk fat fraction was carried out by LTQ-Orbitrap Velos shotgun proteomics on samples from healthy and mastitic animals. A vast array of proteins involved in NET formation and activity were identified, including PADs. Citrullination was evidenced by tandem mass spectrometry in histones released in milk by sheep neutrophils. This work describes the in vivo formation of NETs in the mammary gland of sheep suffering bacterial infection, confirms their association with the MFG fraction, and provides the largest database of proteins expressed in mastitic sheep milk.

## Materials and methods

### Milk and tissue samples

Sheep samples were retrieved from a repository generated in the course of an experimental infection of sheep with *S. uberis*, reported in a previous work [24]. Briefly, Sarda sheep in mid-lactation with no history of mammary infections were subjected to two rounds of infection by intramammary infusion with *S. uberis* in one half-udder (1 × 10^6^ followed by 2 × 10^7^ CFU after six days). Sheep were clinically monitored and milked along the whole infection, and developed an acute clinical mastitis in the infected half-udder. After a total of 12 days, sheep were sacrificed, mammary tissues were collected, and immediately stored at–80 °C. A portion of the sample was also fixed in formalin and embedded in paraffin, and subjected to histopathological examination and grading [24]. For this study, we used milk samples collected from three animals at the at the 3^rd^ day of an experimental mammary infection with 2 × 10^7^ CFU of *S. uberis*, and mammary tissue samples from the same three animals, collected at necroscopy. Milk collected from the same half-udder before infection was used as a control for the normal milk proteome.

### Fluorescent in-situ hybridization and confocal fluorescent immunomicroscopy

Biotin-conjugated specific probes were used for in-situ detection of *S. uberis*, as described previously [24]. Briefly, sections were mounted on slides, deparaffinated, and rehydrated. Pepsin digestion was carried out, sections were post-fixed with an ascending graded series of alcohol, and air-dried. The sections were then incubated in succession with prehybridization solution and hybridization solution, including appropriate washing and blocking steps. The signal was revealed with Streptavidin Alexa Fluor® 555 conjugate (Invitrogen). For immunomicroscopy, the slides obtained from fixed mammary tissues were processed also as described previously [24]. The following antibodies were used: FITC-labeled anti-Neutrophil [7/4] (Abcam Cambridge, MA, USA), anti-S100A9 (Sigma-Aldrich, St. Louis, MO, USA), anti-CAMP (cathelicidin), and anti-histone H4 (Sigma-Aldrich). Alexa Fluor® conjugated secondary antibodies (Invitrogen, Carlsbad, CA, USA) were used for detection. Nuclei were counterstained with the Hoechst stain (Sigma). SYTOX Orange staining was carried out on the milk fat fraction according to Reinhardt et al. [23] Milk was centrifuged at 5000 × *g* for 15 min at 4 °C, and the cream layer was separated, washed twice in phosphate saline buffer, and once in mQ H_2_O at 37 °C for 10 min in slow agitation. Milk fat globules were resuspended in room temperature reaction buffer (10 mM Tris, 150 mM KCl, 250 mM sucrose, 2 mM MgCl_2_, pH 7.5). An aliquot of each sample was treated with 10 units of Benzonase® (Sigma Aldrich) for 30 min at room temperature. Samples were washed twice with reaction buffer and stained with 5 μM SYTOX Orange (Life technologies) for 10 min at room temperature. After staining, samples were washed twice with reaction buffer and 50 μL of resuspended samples were placed on Lab-Tek® Chamber Slides. For tissues, two sections of each sample were deparaffinized and rehydrated following standard histological procedures. Sections were stained with 5 μM SYTOX Orange for 10 min at room temperature. Replicate sections were treated with 10 units of Benzonase® for 30 min at room temperature before staining. A Leica TCS SP 5 confocal microscope (Leica Microsystems, Germany) was used for image acquisition. Processing was made with the LAS AF Lite (Leica Microsystems) for contrast and brightness adjustment.

### Sample preparation by the FASP method

Proteins were extracted from the fat layer fraction as described previously [26-28]. Briefly, milk samples were centrifuged to separate the cream fraction, which was then washed twice in phosphate-buffered saline and once in triple-distilled water. The washed cream was crystallized at 4 °C overnight, and then mechanically homogenized and heated in order to separate the protein fraction from the lipid fraction. Protein extracts were finally quantified with the 2-D Quant kit (GE Healthcare, Uppsala, Sweden) and processed into peptides according to the “FASP II” protocol for generation of tryptic digests [29-31].

### LTQ-Orbitrap analysis of peptides

LC–MS/MS analyses were carried out on an LTQ-Orbitrap Velos (Thermo Scientific, San Jose, CA, USA) interfaced with an UltiMate 3000 RSLCnanoLC system (Dionex, Sunnyvale, CA, USA, Thermo Scientific), as described previously, with minor modifications [32]. Specifically, the peptide mixture was concentrated and washed onto a trapping precolumn (Acclaim PepMap C18, 75 m × 2 cm nanoViper, 3 m, 100 Å, Dionex) and fractionated onto a C18 RP column (Acclaim PepMap RSLC C18, 75 m × 15 cm nanoViper, 2 m, 100 Å, Dionex). The peptide mixture was separated using a linear gradient of eluent B (0.2% formic acid in 95% ACN) in eluent A (0.2% formic acid in 5% ACN) from 10 to 30% in 485 min. Two technical replicates were run for each experimental sample. The fragmentation method was conducted utilizing Higher Energy Collisional Dissociation (HCD) and nitrogen as the collision gas. Proteome Discoverer (version 1.4; Thermo Scientific, Bremen, Germany) using an in-house Mascot server (version 2.3, Matrix Science) was used for protein identification according to the following criteria: Database UniProtKB/Swiss-Prot (release 2015 02), enzyme trypsin; up to two missed cleavages allowed; taxonomy mammalian; precursor mass tolerance 10 ppm; MS/MS tolerance 0.02 Da; charge states +2, +3, and +4; cysteine carbamidomethylation as static modification; asparagines or glutamine deimination, arginine deimination; N-terminal glutamine conversion to pyro-glutammic acid and methionine oxidation as dynamic modifications. The percolator algorithm was used for protein significance and for peptide validation (FDR < 0.01%). Peptide and protein grouping according to the Proteome Discoverer’s algorithm were allowed, applying the strict maximum parsimony principle. Putatively citrullinated fragmentation spectra indicated by Proteome Discoverer were carefully verified manually to remove false assignments.

### Label-free quantitation and statistical evaluation

Spectral counts (SpC) were used as a semiquantitative measure to evaluate protein abundance and to compare protein expression in healthy and mastitic samples, as described previously [24]. Only the proteins with the highest number of unique peptides and SpCs were selected between different homologue proteins present in each sample. Proteins for differential analysis were those having SpC ≥ 2 and present in at least two replicates of the same group. Before differential analysis, the average SpC value was calculated for each protein between replicate samples. The SpC log ratio (R_SC_) was used to express protein abundance and the fold change between different conditions, respectively. R_SC_ were calculated according to the methods described by Old et al., and Zybailov et al., respectively [33,34]. The beta-binomial test was performed to identify differentially expressed proteins according to the method described by Pham et al. [35]. *P* values were corrected by the FDR. Protein localization ontology and protein function ontology classes were determined according to the Universal Protein Resource (UniProt).

## Results

### Confocal microscopy supports formation of NETs in vivo in mammary alveoli of acutely mastitic sheep

**Figure 1:**
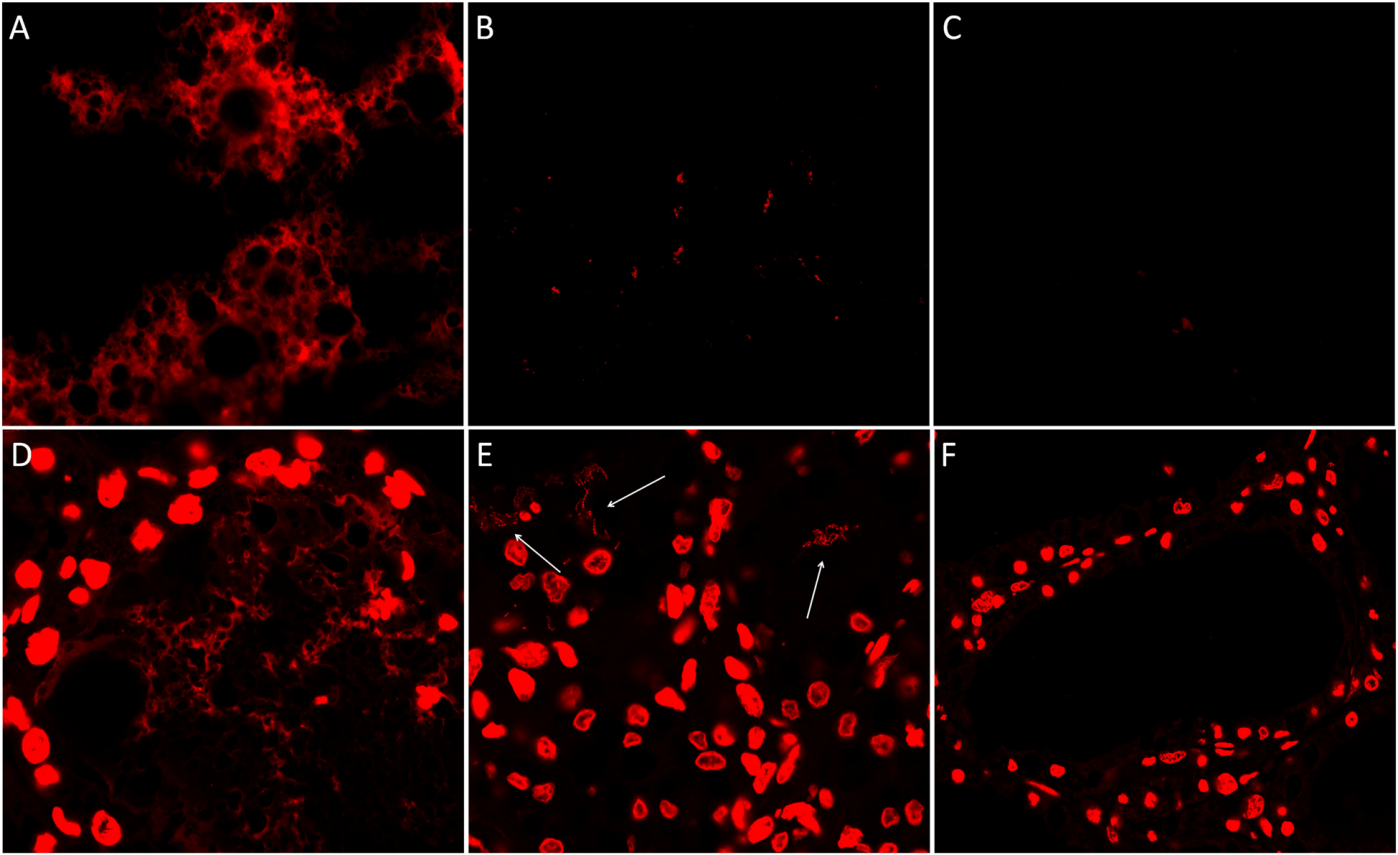
**SYTOX Orange staining of mammary sheep tissues and milk.** **A**. Milk from sheep infected with *S. uberis*. **B**. Same sample as A, after benzonase treatment. **C**. Milk collected before infection. **D**. Tissue from sheep infected with *S. uberis*. **E**. Same as D, after benzonase treatment. The arrows indicate the residual staining of bacteria, now visible after digestion of the extracellular DNA in NETs. **F**. Mammary tissues collected from a healthy animal. Even when a strong contrast is applied to the image, no extranuclear DNA can be seen inside the mammary alveolus.

**Figure 2:**
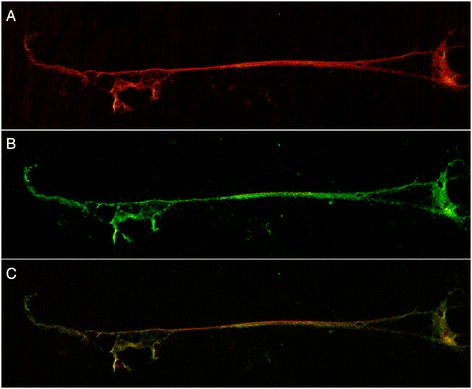
**Colocalization of extranuclear DNA filaments with histones.** SYTOX Orange and histone H4 staining of milk from sheep infected with *S. uberis*. A long stretch of DNA can be seen extruding from a spherical formation on the right. **A**. SYTOX Orange staining. **B**. Immunomicroscopy with anti-histone H4 antibodies. **C**. Overlay image.

Milk and tissues collected at necroscopy from the mammary gland of sheep that developed acute mastitis after experimental *S. uberis* infection were analyzed microscopically by SYTOX Orange staining. As illustrated in Figure 1A, the fat fraction of milk from mastitic sheep showed a brightly stained, three-dimensional DNA structure, characterized by a pronounced and interconnected meshwork of DNA fibers. This structure was almost completely destroyed by treatment with nuclease (Figure 1B), and it was never observed in negative samples (Figure 1C). When subjected to the same staining, mammary tissues from mastitic animals showed a similarly structured DNA network, characterized by the same alveolated, highly reticulated pattern (Figure 1D). This structure disappeared upon nuclease treatment, leaving behind the bead-like chains of stained streptococci that became more clearly visible after digestion of the DNA net (Figure 1E, white arrows). Mammary tissues from healthy animals were always devoid of such structures, even when applying a high contrast to the microscopy images (Figure 1F). The nature of these milk formations as NETs was assessed by colocalization with histones and other NET-associated proteins. Figure 2 shows the results of immunomicroscopy carried out on milk NETs stained with SYTOX Orange (Figure 2A) and anti-histone H4 antibodies (Figure 2B), demonstrating that the DNA formations are associated with histones, one of the hallmark of NETs. The colocalization of the two signals is shown in Figure 2C. In these images, a long stretch of extracellular DNA (red) can be seen extruding from a seemingly spherical formation, showing a clear diffused granular staining with anti-histone H4 antibodies (green), with weblike projections at the extremities.

**Figure 3:**
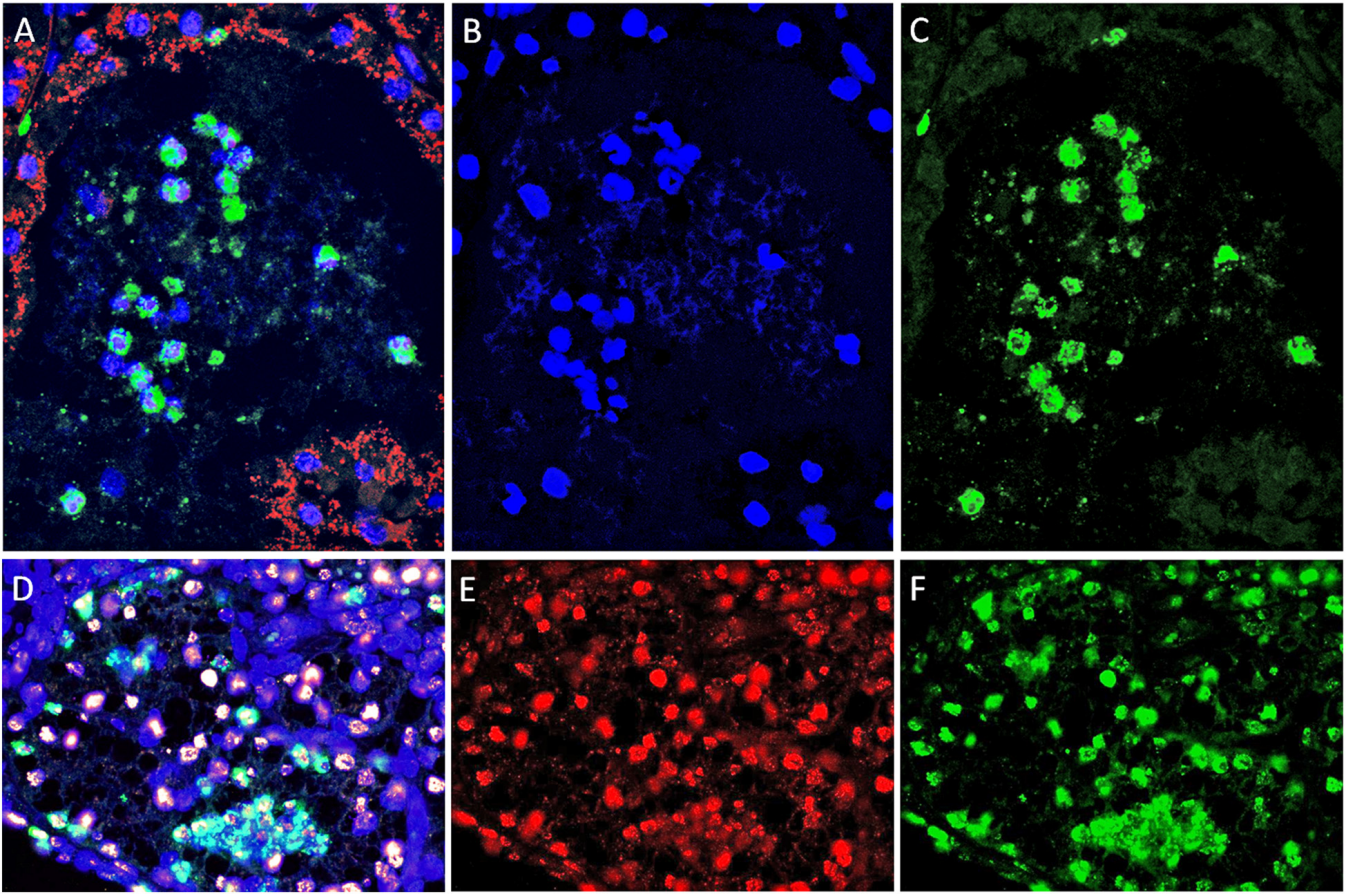
**Immune colocalization and FISH on mastitic mammary sheep tissue.** **A**. Overlay of FISH, Hoechst staining and immunomicroscopy for *S. uberis* (red), DNA (blue), and S100A9 (green). **B**. DNA **C**. S100A9. **D**. Overlay of Hoechst staining (blue) and immunomicroscopy for histone H4 (red), and cathelicidin (green). **E**. Histone H4. **F**. Cathelicidin.

Then, in order to further investigate NETs in mammary alveoli, tissue samples were examined by combined immunocolocalization, fluorescence in-situ hybridization (FISH), and DNA staining for detection of antimicrobial proteins (S100A9 and cathelicidin), histones (histone H4), bacteria (*S. uberis*), and DNA, respectively. The results are summarized in Figure 3. Figure 3A shows mammary alveoli lined with bacteria (red signals) and filled with cell clusters, with extranuclear DNA (blue) interspersed among stained nuclei and colocalizing with S100A9 (green); S100A9 signals were present in epithelial cells lining the alveoli but were stronger within and surrounding the clusters of nuclei inside the lumen. These cell infiltrates were mainly represented by neutrophils, as clearly demonstrated in our previous study on the same tissues [24]. The DNA net among neutrophil nuclei can be seen in the detail shown in Figure 3B, where the blue signal is isolated. The S100A9 signal, isolated in Figure 3C, clearly colocalized with extranuclear DNA. In addition, confocal immunomicroscopy carried out with anti-histone H4 antibodies and cathelicidin, together with the expected nuclear staining, highlighted the colocalization of extranuclear chromatin with cathelicidin (Figure 3D). The immunomicroscopy image illustrates DNA structures (blue) colocalizing with both histone H4 (red) and cathelicidin (green). Histone H4 and cathelicidin signals are also shown isolated in Figure 3E and Figure 3F. Mammary tissues from the uninfected control animal were always devoid of intramammary neutrophils (as shown in Figure 1F) and were negative for antimicrobial proteins. Immunomicroscopy results, together with the preliminary data emerging from our previous investigations [24,25], prompted us to carry out an in-depth proteomic study of mastitic milk to search for proteins and their modifications, with the aim of reinforcing these evidences of NETs in sheep mammary alveoli.

### Localization and function ontologies of differential proteins are consistent with the presence of NETs in mastitic milk

Previous studies carried out in bovines demonstrated that NETs are associated to the milk fat globule (MFG) fraction [23], as also suggested in sheep by our previously reported data [24,25]. Therefore, proteins extracted from the fat layer were investigated in this study. LTQ-Orbitrap shotgun proteomics of this milk fraction produced 1095 valid unique protein identifications (Additional file 1) (filtered from an original number of 2447 total protein identifications, as reported in Additional file 2). All proteins were assessed for their differential abundance in healthy and mastitic animals by means of a label-free quantitation approach (Additional file 3). In total, 488 proteins showed a statistically significant (*P* < 0.05) difference in abundance of at least 1.5 R_SCs_; 201 were higher in healthy milk, and 287 were higher in mastitic milk, respectively (Additional file 4).

Then, an ontology analysis according to localization and function was carried out. The most increased localization class, according to Uniprot, was the cytoplasmic granule, followed by the nucleus and by the mitochondrion. All these classes are coherent with the NET composition, and are in agreement to what reported in the literature on the subject. In fact, NETs are mainly formed by DNA and histones (nucleus) decorated with proteases and antimicrobial proteins from the cytoplasmic granules [16]. Concerning mitochondrial proteins, their increase is also not surprising, since mitochondria are reported to contribute to NET formation [36,37]. The proteins belonging to these categories and undergoing an increase in mastitic milk are listed in Table 1.

**Table 1:** Proteins in localization ontology classes showing an increase in mastitic milk

Accession	Subcellular localizations and protein names	R_SC_
	*Cytoplasmic granule*	
Q29477	Lactotransferrin	4.33
Q8VC88	Grancalcin	2.97
Q9GL30	Phospholipase B-like 1	2.95
Q9UM07	Protein-arginine deiminase type-4	2.38
Q9Y2Q0	Probable phospholipid-transporting ATPase IA	1.57
	*Nucleus*	
P70615	Lamin-B1	5.10
O75367	Core histone macro-H2A.1	5.03
P84227	Histone H3.2	4.83
P0C0S4	Histone H2A.Z	4.65
P16401	Histone H1.5	4.34
P52272	Heterogeneous nuclear ribonucleoprotein M	4.18
P62803	Histone H4	4.00
A7VJC2	Heterogeneous nuclear ribonucleoproteins A2/B1	3.78
O35737	Heterogeneous nuclear ribonucleoprotein H	3.71
Q92841	Probable ATP-dependent RNA helicase DDX17	3.71
Q28141	ATP-dependent RNA helicase A	3.61
Q5E9J1	Heterogeneous nuclear ribonucleoprotein F	3.54
Q8NF91	Nesprin-1	3.48
P40673	High mobility group protein B2	3.33
P07156	High mobility group protein B1 (Fragment)	3.16
Q8BJS4	SUN domain-containing protein 2	3.13
P29350	Tyrosine-protein phosphatase non-receptor type 6	3.11
Q8WN55	Polypyrimidine tract-binding protein 1	3.09
P02252	Histone H1.4	3.07
P11387	DNA topoisomerase 1	3.01
P46193	Annexin A1	2.93
Q92522	Histone H1x	2.86
P32120	Beta-arrestin-2	2.85
P13084	Nucleophosmin	2.84
P27214	Annexin A11	2.81
P51991	Heterogeneous nuclear ribonucleoprotein A3	2.71
Q13838	Spliceosome RNA helicase DDX39B	2.66
Q96KK5	Histone H2A type 1-H	2.66
P43243	Matrin-3	2.61
Q3T149	Heat shock protein beta-1	2.55
Q6AXS3	Protein DEK	2.54
Q2HJ57	Coactosin-like protein	2.48
P04256	Heterogeneous nuclear ribonucleoprotein A1	2.41
Q9CW03	Structural maintenance of chromosomes protein 3	2.41
P23196	DNA-(apurinic or apyrimidinic site) lyase	2.40
Q9UM07	Protein-arginine deiminase type-4	2.38
Q15233	Non-POU domain-containing octamer-binding protein	2.35
Q60710	SAM domain and HD domain-containing protein 1	2.35
Q3T094	Protein ETHE1, mitochondrial	2.32
P38919	Eukaryotic initiation factor 4A-III	2.30
Q8CCK0	Core histone macro-H2A.2	2.27
Q9H8H2	Probable ATP-dependent RNA helicase DDX31	2.27
P62318	Small nuclear ribonucleoprotein Sm D3	2.26
Q0VCY7	Serine/arginine-rich splicing factor 1	2.11
Q3ZBV3	Protein mago nashi homolog	2.04
Q64399	DNA topoisomerase 2-beta	2.04
Q6P2Q9	Pre-mRNA-processing-splicing factor 8	1.97
Q03252	Lamin-B2	1.90
P05126	Protein kinase C beta type	1.89
A0JN52	Splicing factor 3B subunit 3	1.89
Q00PI9	Heterogeneous nuclear ribonucleoprotein U-like protein 2	1.89
Q3SZF8	Small nuclear ribonucleoprotein Sm D2	1.89
P60122	RuvB-like 1	1.87
P47845	Galectin-3	1.85
O35286	Putative pre-mRNA-splicing factor ATP-dependent RNA helicase DHX15	1.83
Q3T0D0	Heterogeneous nuclear ribonucleoprotein K	1.81
Q5EA36	RNA-binding protein 14	1.80
Q13247	Serine/arginine-rich splicing factor 6	1.69
P38646	Stress-70 protein, mitochondrial	1.66
A5PJZ5	Nuclear pore complex protein Nup93	1.65
Q06A98	Serine/arginine-rich splicing factor 2	1.62
P02545	Prelamin-A/C	1.57
Q5PXY7	Cellular retinoic acid-binding protein 2 O	1.54
	*Mitochondrion*	
P45879	Voltage-dependent anion-selective channel protein 1	4.74
P20000	Aldehyde dehydrogenase, mitochondrial	3.90
Q9Y6N5	Sulfide:quinone oxidoreductase, mitochondrial	3.69
Q9MZ13	Voltage-dependent anion-selective channel protein 3	3.43
Q9UKU0	Long-chain-fatty-acid-CoA ligase 6	3.26
P41976	Superoxide dismutase [Mn], mitochondrial	3.17
Q3T165	Prohibitin	3.11
Q05B71	CDGSH iron-sulfur domain-containing protein 2	2.91
P11024	NAD(P) transhydrogenase, mitochondrial	2.83
P31081	60 kDa heat shock protein, mitochondrial	2.55
Q9BGI1	Peroxiredoxin-5, mitochondrial	2.46
Q29RK1	Citrate synthase, mitochondrial	2.40
P23196	DNA-(apurinic or apyrimidinic site) lyase	2.40
P48818	Very long-chain specific acyl-CoA dehydrogenase, mitochondrial	2.32
Q3T094	Protein ETHE1, mitochondrial	2.32
P23004	Cytochrome b-c1 complex subunit 2, mitochondrial	2.18
Q2HJ97	Prohibitin-2	2.16
Q8WY22	BRI3-binding protein	2.12
P00829	ATP synthase subunit beta, mitochondrial	2.03
Q28852	ATP synthase subunit g, mitochondrial	1.99
A5PJZ1	Calcium-binding mitochondrial carrier protein SCaMC-1	1.85
P05631	ATP synthase subunit gamma, mitochondrial	1.81
P13620	ATP synthase subunit d, mitochondrial	1.79
P23709	NADH dehydrogenase [ubiquinone] iron-sulfur protein 3, mitochondrial	1.72
P31039	Succinate dehydrogenase [ubiquinone] flavoprotein subunit, mitochondrial	1.69
P38646	Stress-70 protein, mitochondrial	1.66
P00423	Cytochrome c oxidase subunit 4 isoform 1, mitochondrial	1.58
Q3ZBI7	Up-regulated during skeletal muscle growth protein 5	1.52

All differences were statistically significant (*P* < 0.05; Beta-binomial test).

In terms of protein functions, numerous proteins that were highly abundant in milk from mastitic animals fell into classes related to immune defence or antimicrobial activities, and are listed in Table 2. Also in this case, protein function classes such as “secretory granule”, “protein citrullination”, “neutrophil chemotaxis”, and “peptidase activity” were strongly supportive of the presence of NETs. Considering that NETs are a recently described immune process, and that functional annotation in this respect is not complete, classical ontology analysis was integrated with a search for NET markers in the scientific literature. As a result of this analysis, almost all the reported NET markers were found to be increased in mastitic milk, and are listed in Table 3 [17,20,23,38].

**Table 2:** Selected protein function ontology classes including proteins involved in inflammation and immune response processes

Accession	Protein functions and names	R_SC_
	*Defense response*	
P05164	Myeloperoxidase	5.62
B6E141	Haptoglobin (Zonulin)	4.78
O77774	Neutrophil cytosol factor 1	4.70
P0C0S4	Histone H2A.Z	4.65
P54230	Cathelicidin-1	4.09
P70248	Unconventional myosin-If	3.79
Q00655	Tyrosine-protein kinase SYK	3.64
P50415	Cathelicidin-3	3.57
P06800	Receptor-type tyrosine-protein phosphatase C	3.07
P80190	Lysozyme C, kidney isozyme	3.07
P56425	Cathelicidin-7	2.85
Q2HJ57	Coactosin-like protein	2.48
P79362	Cathelicidin-2	2.46
Q60710	SAM domain and HD domain-containing protein 1	2.35
P17453	Bactericidal permeability-increasing protein	2.33
Q92608	Dedicator of cytokinesis protein 2	2.30
P80025	Lactoperoxidase	1.50
	*Immune response*	
P70248	Unconventional myosin-If	3.79
P15497	Apolipoprotein A-I	2.10
Q29477	Lactotransferrin	4.33
Q00655	Tyrosine-protein kinase SYK	3.64
O46521	Cytochrome b-245 light chain	3.12
Q3UP87	Neutrophil elastase	2.77
Q3MHR7	Actin-related protein 2/3 complex subunit 2	3.39
Q92608	Dedicator of cytokinesis protein 2	2.30
P06800	Receptor-type tyrosine-protein phosphatase C	3.07
Q60710	SAM domain and HD domain-containing protein 1	2.35
P13796	Plastin-2	2.30
P47845	Galectin-3	1.85
P11215	Integrin alpha-M	1.55
	*Innate immune response*	
Q29477	Lactotransferrin	4.33
P70248	Unconventional myosin-If	3.79
Q00655	Tyrosine-protein kinase SYK	3.64
Q3MHR7	Actin-related protein 2/3 complex subunit 2	3.39
O46521	Cytochrome b-245 light chain	3.12
P28783	Protein S100-A9	2.79
P28782	Protein S100-A8	2.65
Q60710	SAM domain and HD domain-containing protein 1	2.35
P47845	Galectin-3	1.85
P11215	Integrin alpha-M	1.55
	*Inflammatory response*	
Q8SPQ0	Chitinase-3-like protein 1	5.09
O77774	Neutrophil cytosol factor 1	4.70
O46521	Cytochrome b-245 light chain	3.12
Q2UVX4	Complement C3	2.85
P28783	Protein S100-A9	2.79
Q3UP87	Neutrophil elastase	2.77
P28782	Protein S100-A8	2.65
P15497	Apolipoprotein A-I	2.10
P98066	Tumor necrosis factor-inducible gene 6 protein	2.08
	*Peptidase activity*	
Q29477	Lactotransferrin	4.33
Q2UVX4	Complement C3	2.85
P28783	Protein S100-A9	2.79
Q3UP87	Neutrophil elastase	2.77
P28782	Protein S100-A8	2.65
P80209	Cathepsin D	2.27
P81286	Plasminogen	2.25
P23004	Cytochrome b-c1 complex subunit 2, mitochondrial	2.18
Q27970	Calpain-1 catalytic subunit	1.85
	*Integrin-mediated signaling pathway*	
Q5VI41	Integrin beta-2	6.53
Q00655	Tyrosine-protein kinase SYK	3.64
P11215	Integrin alpha-M	1.55
	*Neutrophil chemotaxis*	
Q00655	Tyrosine-protein kinase SYK	3.64
P28783	Protein S100-A9	2.79
P28782	Protein S100-A8	2.65
P11215	Integrin alpha-M	1.55
	*Acute-phase response*	
B6E141	Haptoglobin (Zonulin)	4.78
P85521	Scavenger receptor cysteine-rich type 1 protein	2.49
	*Protein citrullination*	
O02849	Protein-arginine deiminase type-3	2.86
Q9UM07	Protein-arginine deiminase type-4	2.38
	*Secretory granule*	
P05164	Myeloperoxidase	5.62
Q3UP87	Neutrophil elastase	2.77

All differences were statistically significant (*P* < 0.05; Beta-binomial test).

**Table 3:** Marker proteins associated with NETs, with their relative spectral counts, identified in mastitic sheep milk [17,20,23,38]

Accession	NET components	Rsc
P17453	Bactericidal/Permeability-increasing protein	2.33
P54230	Cathelicidin-1	1.09
P79362	Cathelicidin-2	2.46
P50415	Cathelicidin-3	3.57
P56425	Cathelicidin-7	2.85
P80209	Cathepsin D	2.27
P02252	Histone H1.4	3.07
P16401	Histone H1.5	4.34
P0C0S4	Histone H2A	4.65
P58876	Histone H2B	3.76
P84227	Histone H3.2	4.83
P62803	Histone H4	3.97
Q29477	Lactoferrin	4.33
P80190	Lysozyme C	3.07
P05164	Myeloperoxidase	5.62
Q3UP87	Neutrophil Elastase	2.77
Q0VCG9	Pentraxin-related protein PTX3	4.05
P28782	Protein S100-A8 (calprotectin L1L subunit)	2.65
P28783	Protein S100-A9 (calprotectin L1H subunit)	2.79

All variations were statistically significant (*P* < 0.001; Beta-binomial test).

### Histones in acutely mastitic sheep milk are citrullinated

One of the biochemical hallmark of NETs is citrullination of histone proteins [39-41]. This modification consists of a deimination of arginine residues, performed by protein-arginine deiminases (PAD1-PAD4), that leads to a shift of 0.985 Da in molecular mass. A very stringent bioinformatic and accurate manual analysis of all peptide fragmentation spectra, required for warranting attribution of this modification, enabled to detect a total of four peptides carrying the arginine to citrulline modification, belonging to histones H1.4, H2A and H2B, as shown in Table 4.

**Table 4:** Citrullinated peptides detected after searching all protein identifications for post-translational modifications

Histone	Peptide Sequence	Position	Replicate	Healthy			Mastitic		
				b	B	C	A	B	C
H1.4	ER*SGVSLAALK	53–63	I	-	-	-	+57	+59	+60
			II	**-**	**-**	**-**	**+**56	**+**44	**+**57
H1.4	ER*SGVSLAALKK	53–64	I	-	-	-	-	-	+53
			II	**-**	**-**	**-**	**-**	**-**	**+**44
H2A	SSR*AGLQFPVGR	19–30	I	**-**	**-**	**-**	**+**42	**+**46	**+**38
			II	**-**	**-**	**-**	**+**32	**+**38	**+**40
H2A	SR*SSR*AGLQFPVGR	17–30	I	**-**	**-**	**-**	**-**	**+**35	**-**
			II	**-**	**-**	**-**	**-**	**+**45	**-**
H2B	STITSR*CIQTAVR	88–100	I	**-**	**-**	**-**	**-**	**+**42	**-**
			II	**-**	**-**	**-**	**-**	**+**38	**-**

All citrullination sites were confirmed by manual verification of peptide mass spectra. For citrullinated peptides, ion scores are reported in the Table.

**Figure 4:**
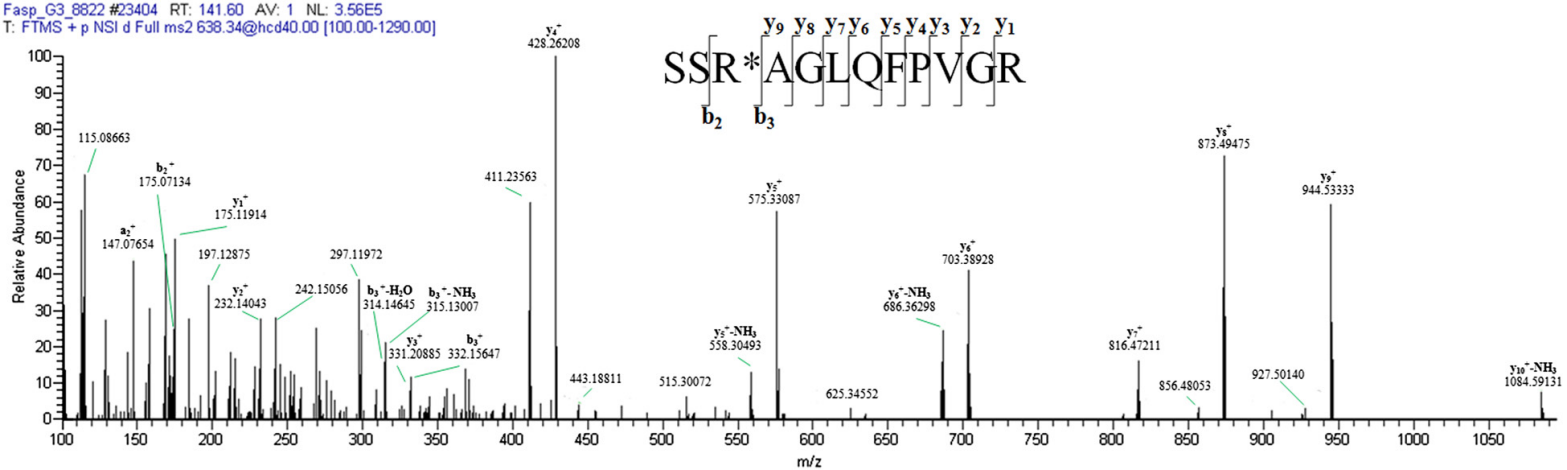
**Representative spectrum of a citrullinated peptide.** The HCD fragmentation spectrum of peptide SSR*AGLQFPVGR, belonging to histone H2A, at *m/z* 638.34351 Da, z = +2, is reported.

Figure 4 reports a representative fragmentation spectrum, relative to the citrullinated peptide SSR*AGLQFPVGR (other citrullinated peptide spectra are reported in Additional file 5). These peptides show a backbone composed by cleavage products of b and y ions and several neutral loss ions, as well as the loss of ammonia (−17 Da) and water (−18 Da) near the precursor ion. In the y ion series all products that lead to modified arginine residue can be seen, highlighting that the presence of this residue disadvantages ionization of subsequent fragments, due to the loss of a positive charge associated with citrullination [42] (Figure 3). Sequence coverage on modified arginine is granted by the products deriving from the b series and neutral loss ions.

## Discussion

In two previous studies carried out by our group on sheep mastitis caused by *M. agalactiae* and *S. uberis* [24,25], aimed at clarifying the role played in vivo by mammary epithelial cells (MECs) in the innate immune response to infection, we detected several proteins that have been recently associated to NETs [17,20,23,38]. In light of these very preliminary findings, the mammary gland tissue and milk of sheep experimentally infected with *S. uberis* were investigated by DNA staining, immune microscopy and FISH. In addition, an in-depth proteomic study was carried out on milk collected before and after infection. With these matched analyses, it was therefore possible to investigate inflamed/infected tissue collected at necroscopy, and therefore to image cells and putative NETs in situ, in vivo, and in the context of inflammation, adding to the information provided by the fluid where NETs and their protein components should be found, that is, milk. As a result, presence of NETs in milk was demonstrated by DNA staining of the extracellular meshwork shown in Figure 1A, that disappeared after nuclease treatment (Figure 1B), as well as by the colocalization (Figure 2) of extracellular DNA filaments with histones. In addition, characterization of mastitic tissue by DNA staining (Figure 1C) and immunomicroscopy for NET markers (Figure 3) highlighted mammary alveoli filled with neutrophils, arranged in clusters within a net showing strong positivity to antimicrobial proteins and extracellular chromatin. In turn, proteomic analysis of milk samples offered the unique opportunity to assess the real changes in protein abundance with a non-targeted approach, highlighting also phenomena not directly dependent from an increase in gene transcription or not directly related to the cells being investigated, as well as to characterize post-translational protein modifications that may play key roles in this pathogenetic process.

The high performance shotgun proteomics approach applied in this work, combining filter-aided sample preparation (FASP), long gradient LC, and LTQ-Orbitrap Velos mass spectrometry, enabled a significant increase in the number of identified proteins when compared to the methods used in our previous studies. In fact, this approach provided a total of 1095 unique protein identifications, significantly higher than the previously available 185 for the study on *M. agalactiae* infection [25] and 849 for the study on *S. uberis* infection [24], making this the largest protein identification database currently available in the scientific literature for mastitic sheep milk, and including 287 proteins significantly increased in mastitis. When examining the list of increased proteins (Additional file 4), an elevated number of these were related to NETs, when compared to the milk of healthy animals. Concerning the localization ontologies of differential proteins, the most significantly increased classes were the cytoplasmic granule, the nucleus and the mitochondrion, in line with the literature findings on NETs (Table 1). In fact, cytoplasmic granule and nucleus proteins are the main components of NETs, being these represented by a DNA mesh with bound histones, proteases, and antimicrobial proteins. Concerning the mitochondrion as the third significantly increased protein localization class, although it might appear surprising, this is actually coherent with presence of NETs. In fact, the release of mitochondrial contents has been reported as another mechanism by which immune cells can form NETs, that is, by the controlled extrusion of mitochondrial DNA without loss of cell viability, although the steps by which this occurs remain poorly characterized [18,36,37].

When proteins were classified according to function, their involvement in immune defence was clearly evident, with all the increased classes belonging to defence response mechanisms (Table 2). It is interesting to notice that, among others, increased proteins include a large amount of proteases, antimicrobial proteins, histones, as well as other proteins possibly related to neutrophil migration and cytoskeletal activity. It is interesting to comment on the detection of haptoglobin, a protein which is well known for its role as a scavenger for heme resulting from haemoglobin degradation. In light of this, the finding of such high amounts in the mastitic udder might appear a bit unusual, although coherent with the numerous reports of haptoglobin increase in serum in presence of mastitis. Interestingly, it has recently been demonstrated that full-length haptoglobin is actually zonulin, that produces the two alpha and beta haptoglobin chains only upon cleavage. Since protein identification by MS relies on detection of tryptic peptide sequences, zonulin peptides will be identified as haptoglobin, since this is the standard annotation for this aminoacid sequence. As zonulin, full-length haptoglobin is long known to increase epithelial permeability, by allowing intercellular tight junction disassembly [43]. In this context, high local concentrations of zonulin may have the function of increasing epithelial permeability to facilitate the influx of immune cells and aid clearance of intramammary infections. The detection of this protein further highlights the power of proteomics in revealing also proteins possibly derived from other cellular sources, or as a result of serum protein leakage into the tissue.

When evaluating function ontology classes, it should be kept in mind that many proteins associated with NETs do not classify as related to immune functions since, being this a recently described immune defence process, functional annotations concerning these proteins are not complete. This problem was also recently highlighted by Reinhardt et al. in their study on bovine mastitis [23]. Therefore, ontology function classes have been integrated with a literature search, and Table 3 reports all known NET markers identified. This table highlights the high abundance of almost all proteins known to be associated to NETs.

An interesting function class identified as significantly increased was “protein citrullination”, including protein-arginine deiminase 4 (PAD4). This protein is thought to be responsible for the key biochemical event triggering NET formation, that is, histone citrullination, that leads to chromatin decondensation followed by rupture of the nuclear membrane and mixing of chromatin with the cytoplasmic granule contents, and then by release of this toxic mixture in the extracellular space. PAD4 is essential for NET formation; in addition, its inhibition decreases NET formation, while its stimulation facilitates the process [19,44]. In light of this observation, we searched for this post-translational modification, and we were able to identify five peptides carrying it, all belonging to histones. Nevertheless, the missed detection of citrullinated histones in healthy animals might be due to the differences in histone abundance existing between the two sample classes. Therefore, this information should be taken only as an indication of the presence of citrullinated peptides in mastitic milk, not as a proof of their differential abundance. Specific, dedicated mass spectrometry experiments will be required to provide a definitive, quantitative measure of this finding.

Our proteomic investigations focused on the MFG fraction, since it has been demonstrated that putative NET proteins in milk are associated to it [23-25]. It remains to be clarified why NETs are so tightly associated to the MFG fraction. It is possible that these vesicles remain trapped in the sticky DNA web formed by NETs, facilitating enrichment of NET components in protein preparations entailing separation of the fat layer. In addition, considering the role played by MECs in the innate immune response of the mammary gland [24,25], we cannot rule out some sort of contribution by epithelial cells in extracellular trap formation [45]. Nevertheless, whether this association somehow influences NET formation and function is yet to be demonstrated.

In conclusion, this study reports the formation of NETs in mammary alveoli of sheep suffering bacterial mastitis, providing detailed information on their composition and opening interesting perspectives for understanding pathogenic mechanisms enacted by bacteria to survive the action of the innate immune system in the mammary gland. In addition, and equally important, this study enlarges the dataset of proteins that increase in milk during bacterial mastitis, supporting the development of new diagnostic strategies for mastitis detection independently from the causal agent. In fact, being these proteins part of the innate immune response, and therefore produced in the course of infection by different pathogenic agents, these can represent the basis for non-specific and universal mastitis detection tools. Even more interestingly, being NETs an innate immune defence mechanism shared also by bovines and, likely, by other dairy ruminants, these findings might be extended to other dairy species of interest, supporting the development of novel and efficient diagnostic tools against this economically impacting disease.

## Additional files

Additional file 1:
**Proteins having the highest number of unique peptides and SpCs among protein homologues.** Only proteins having the highest number of unique peptides per homologue among all those identified are reported in the table. Additional file 2:
**List of all identifications with peptide information.** Protein identifications and detailed peptide information are reported according to the Proteome Discoverer output. Additional file 3:
**Results of the differential analysis for all identified proteins.** All identified proteins are reported together with their respective spectral counts, fold changes, and p values according to the beta-binomial test. Additional file 4:
**Proteins with fold changes ±1.5 and** ***p*** **≤ 0.05.** Only proteins having fold changes ±1.5 and *p* ≤ 0.05 according to the beta-binomial test are reported, together with their respective spectral counts. Additional file 5:
**Citrullinated peptide spectra.** The spectra for citrullinated peptides detected in all samples are reported, together with detailed annotations.
