# Supplementary material for: Neutrophil extracellular traps in sheep mastitis
Source: Vet Res. 2015 Jun 18;46(1):59. doi: 10.1186/s13567-015-0196-x (PMC4471908; doi:10.1186/s13567-015-0196-x)

Mastitic A_Replicate I

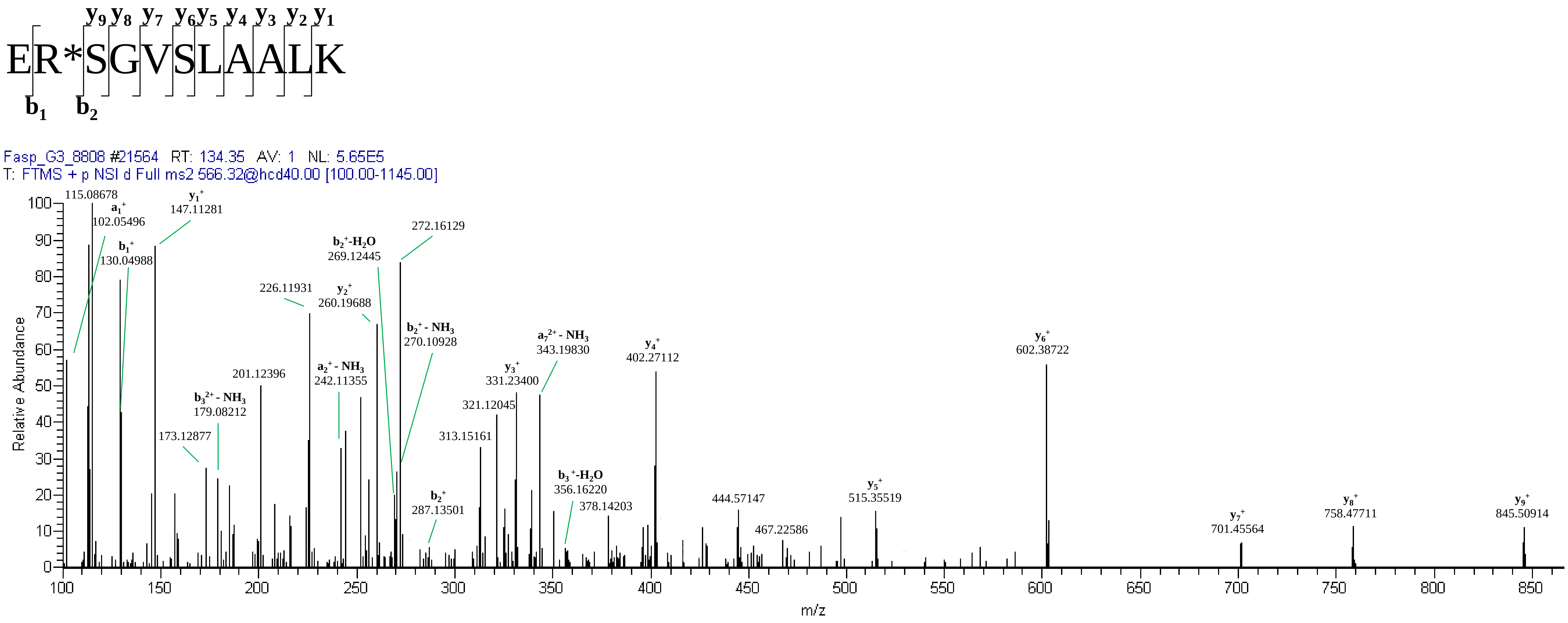

Mastitic A_Replicate II

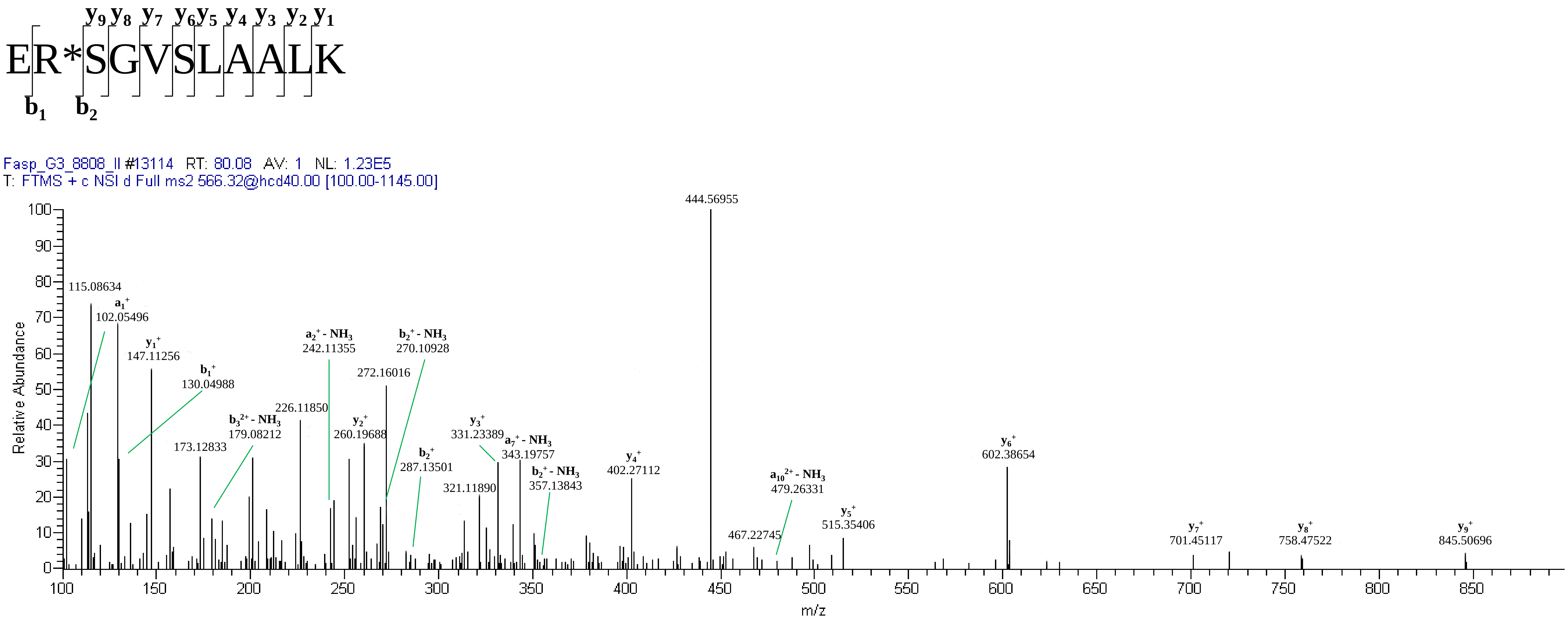

Mastitic B_Replicate I

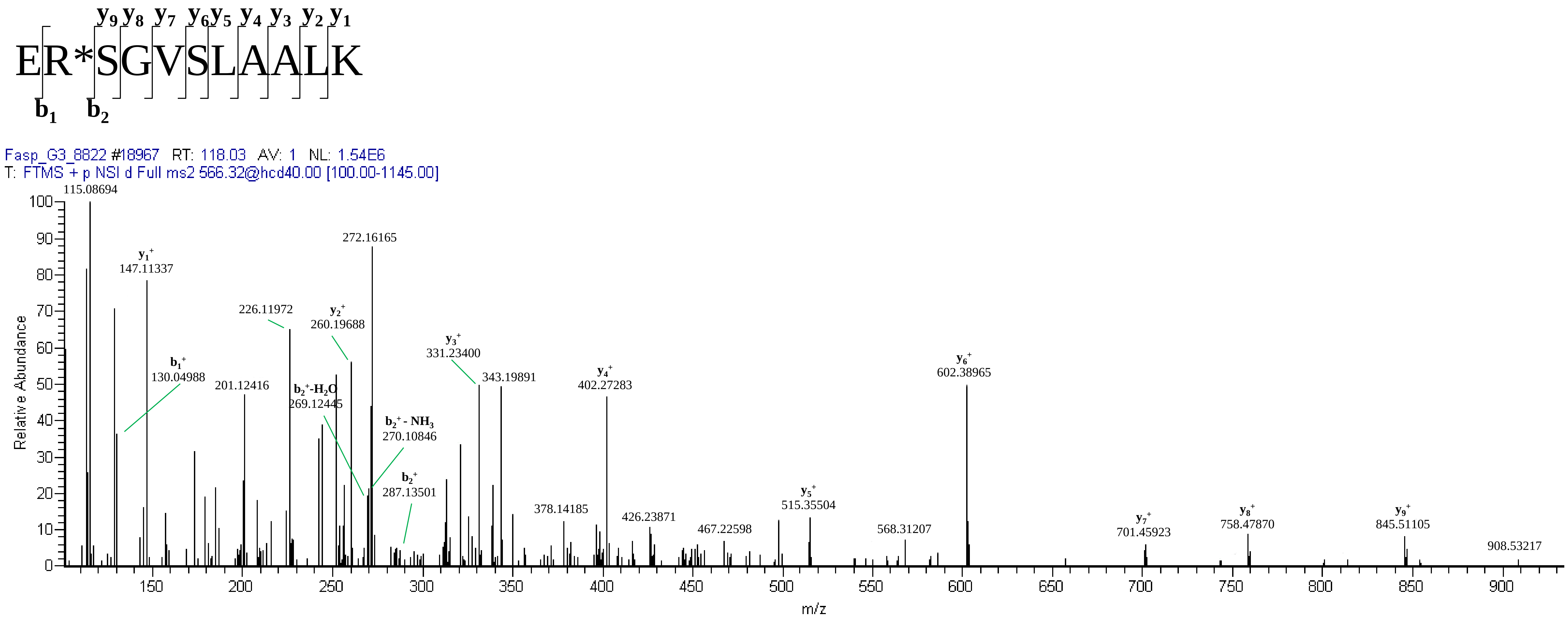

Mastitic B_Replicate II

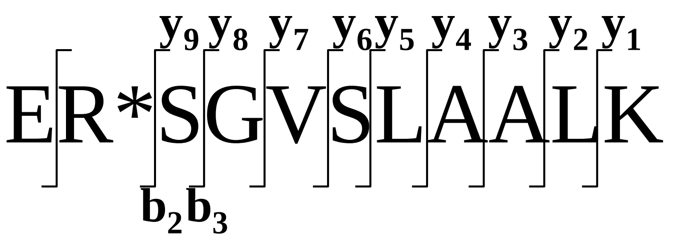

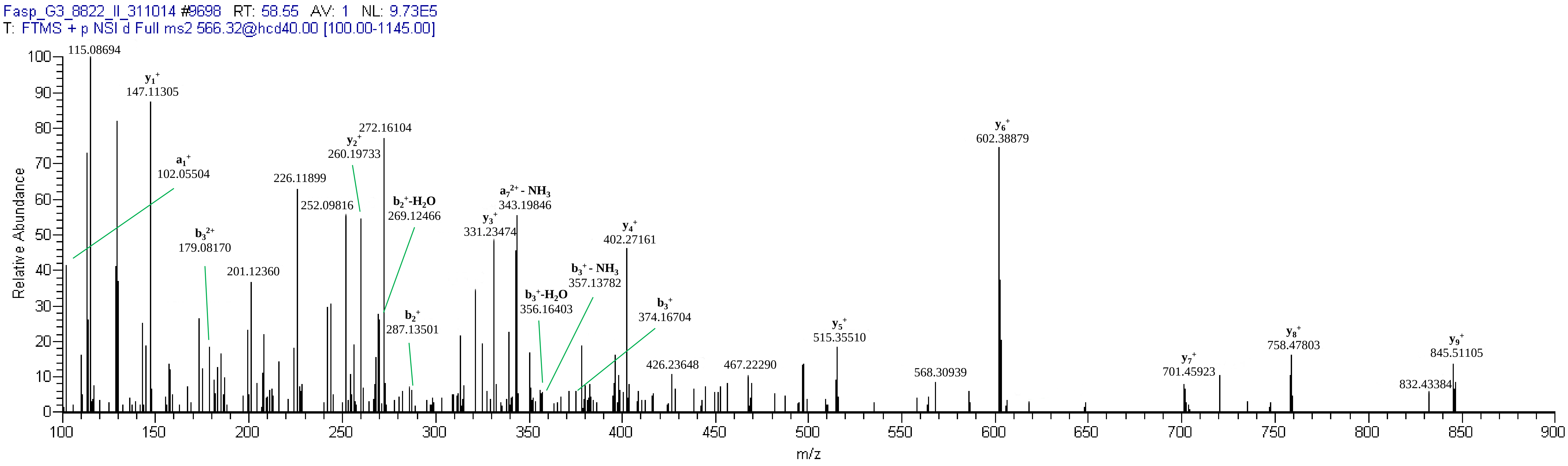

Mastitic C_Replicate I

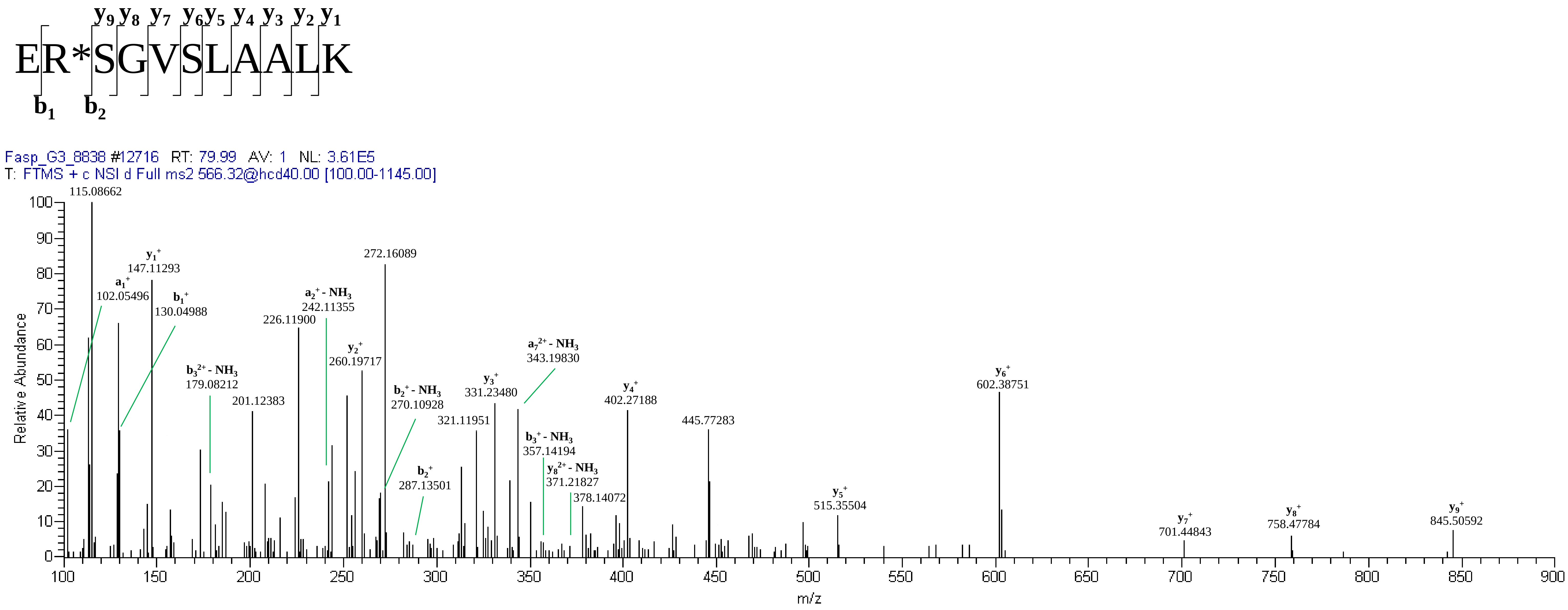

Mastitic C_Replicate II

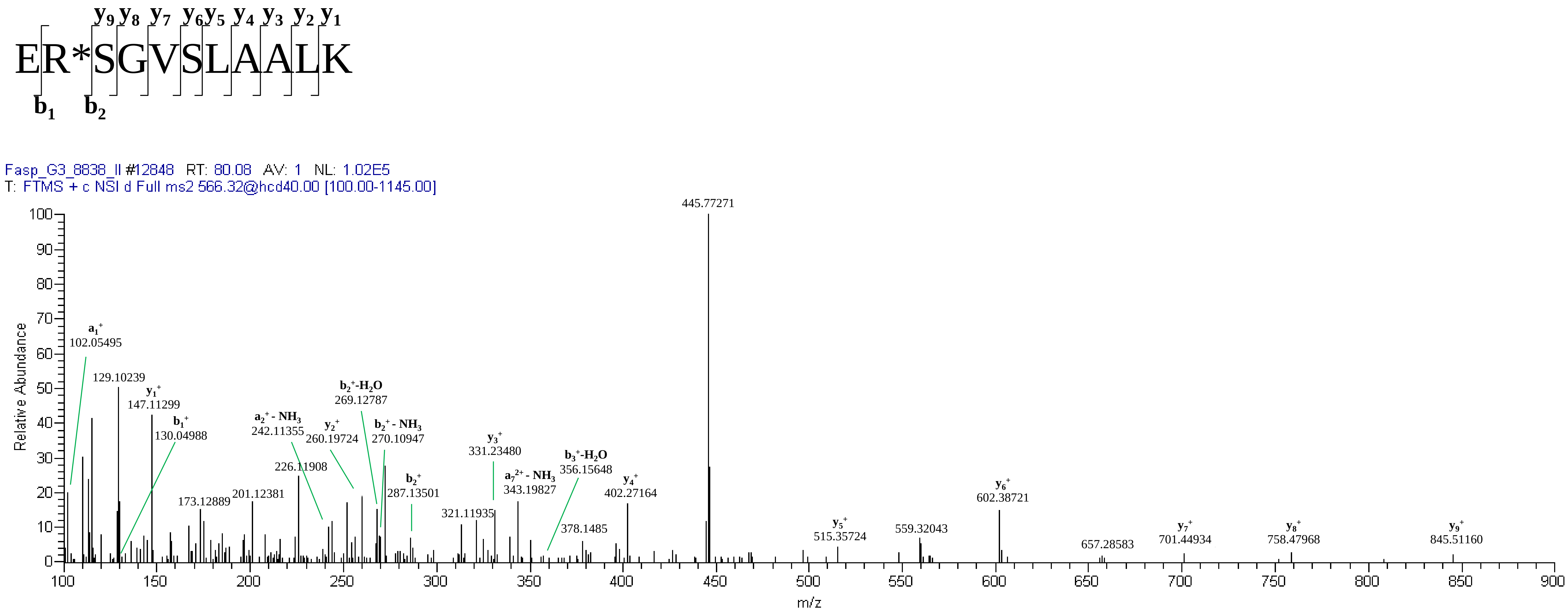

Mastitic C_Replicate I

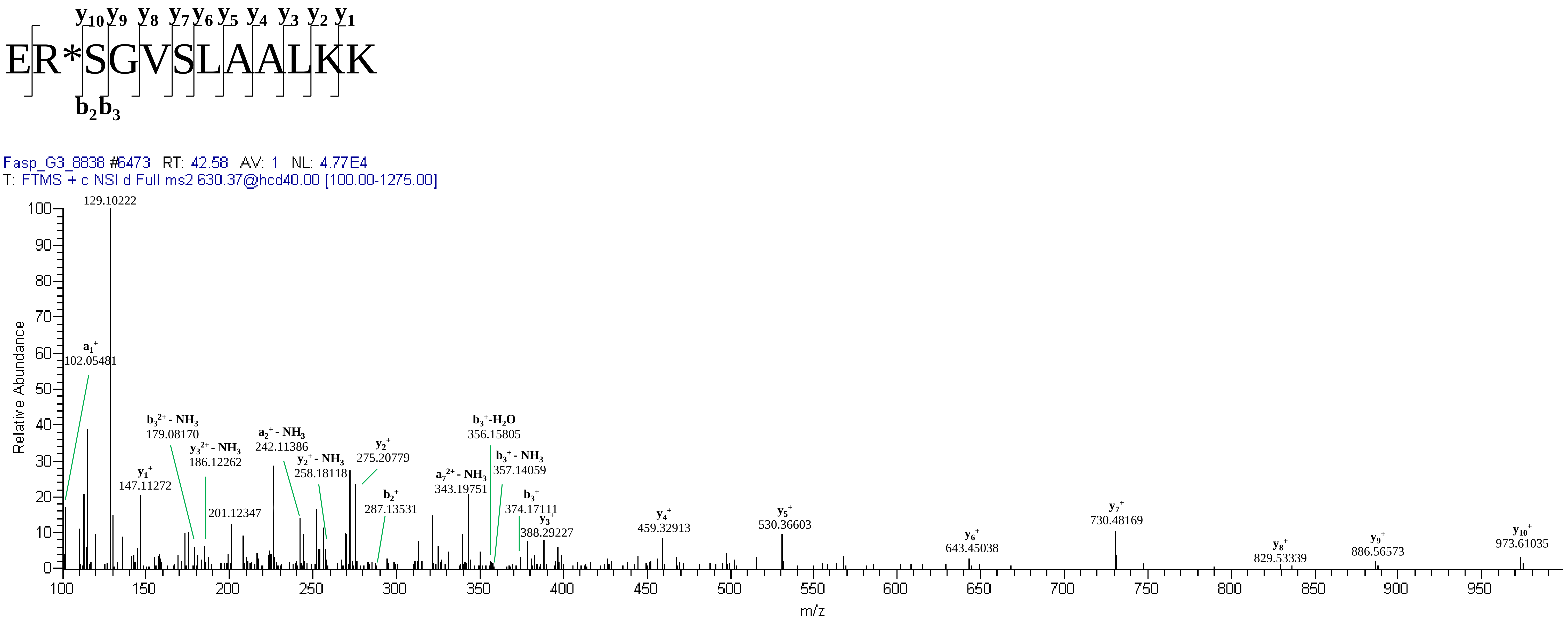

Mastitic C_Replicate II

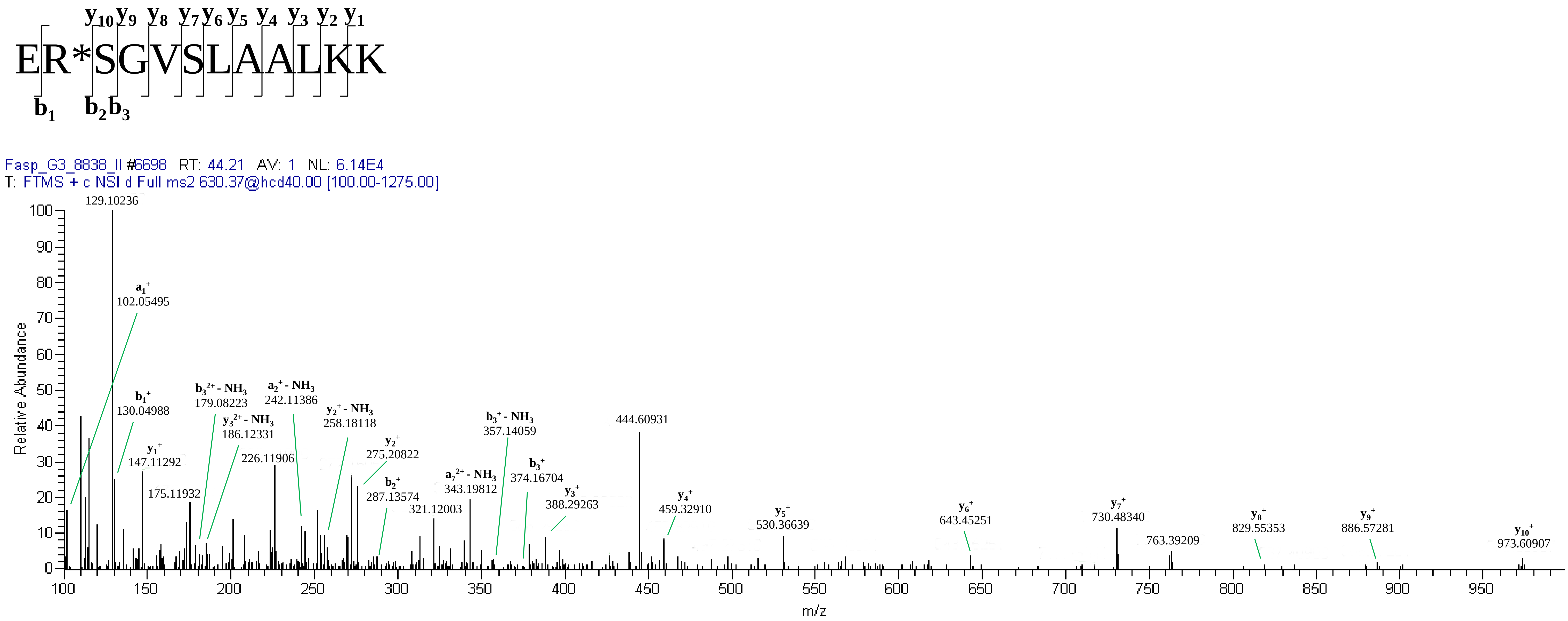

Mastitic A_Replicate I

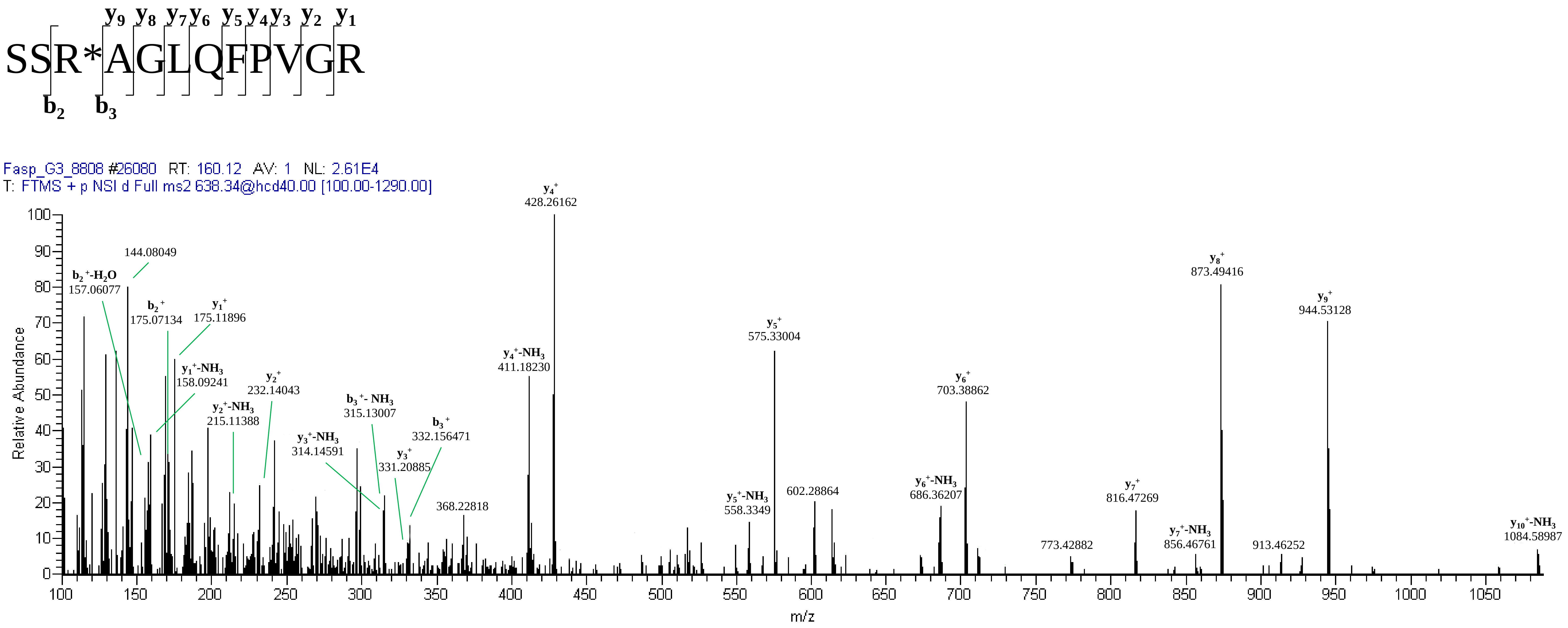

Mastitic A_Replicate II

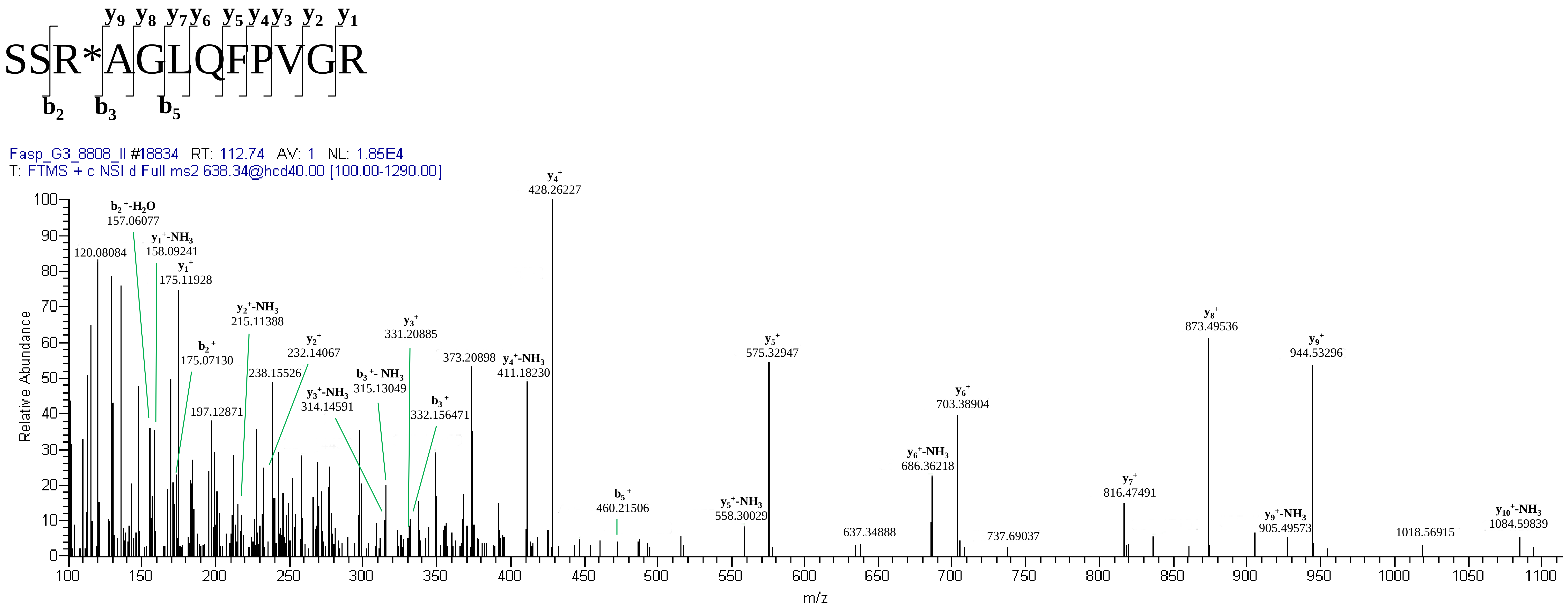

Mastitic B_Replicate I

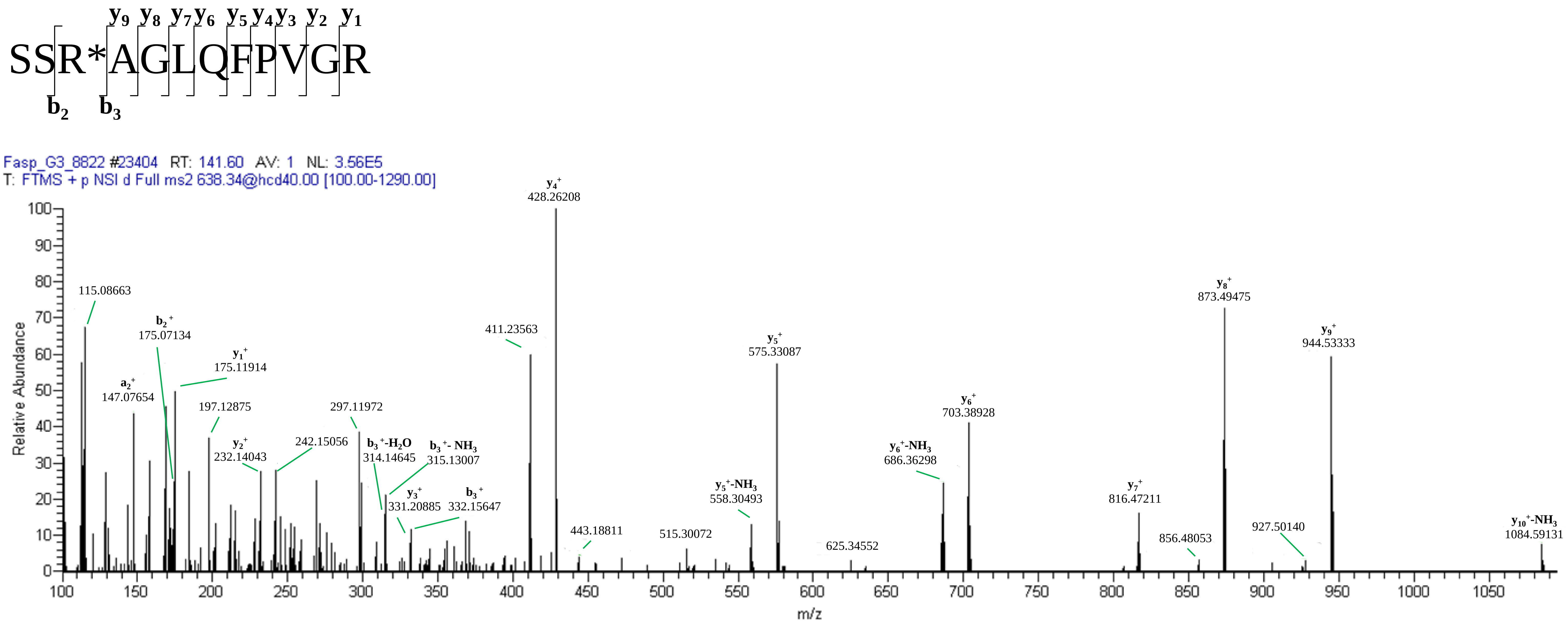

Mastitic B_Replicate II

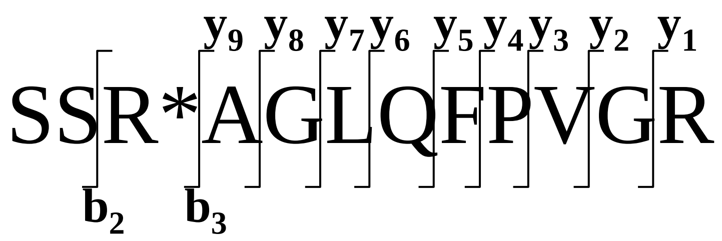

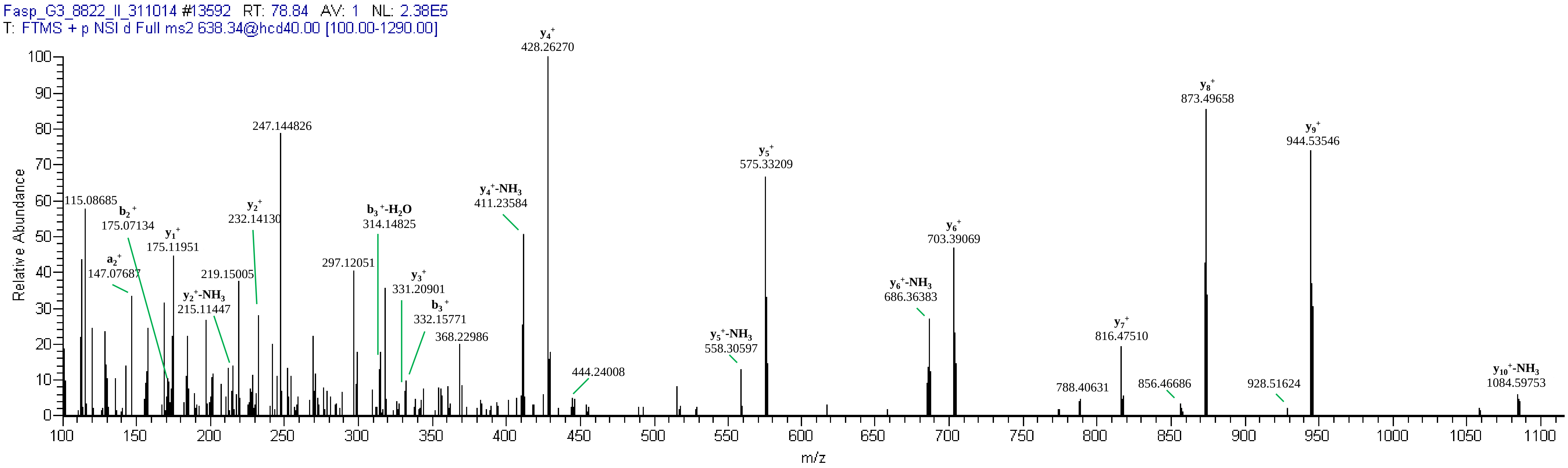

Mastitic C_Replicate I

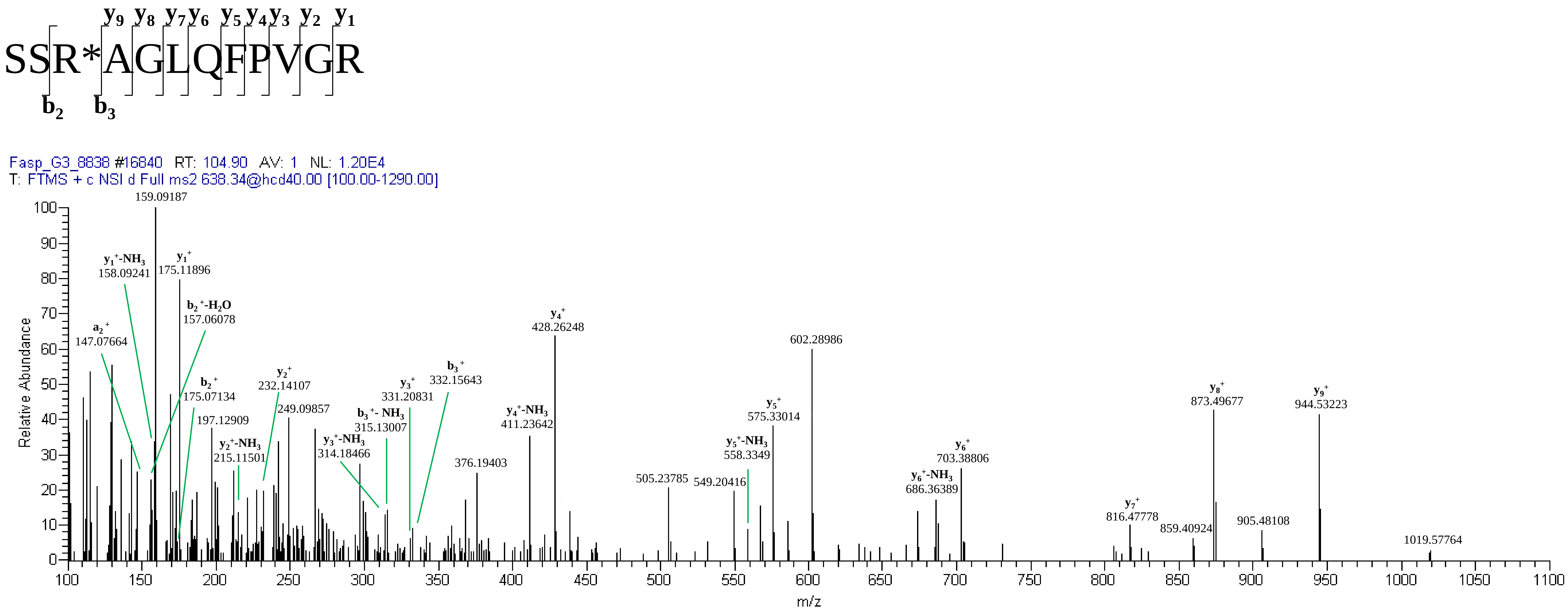

Mastitic C_Replicate II

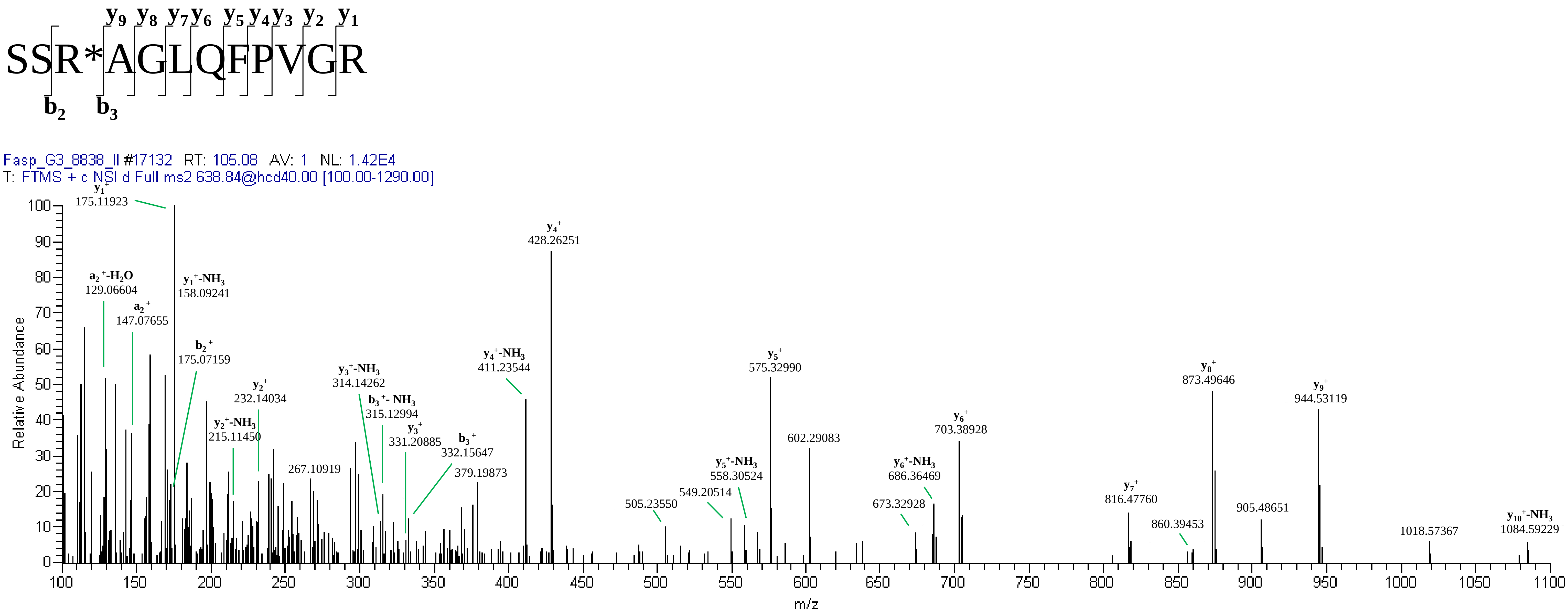

Mastitic B Replicate I

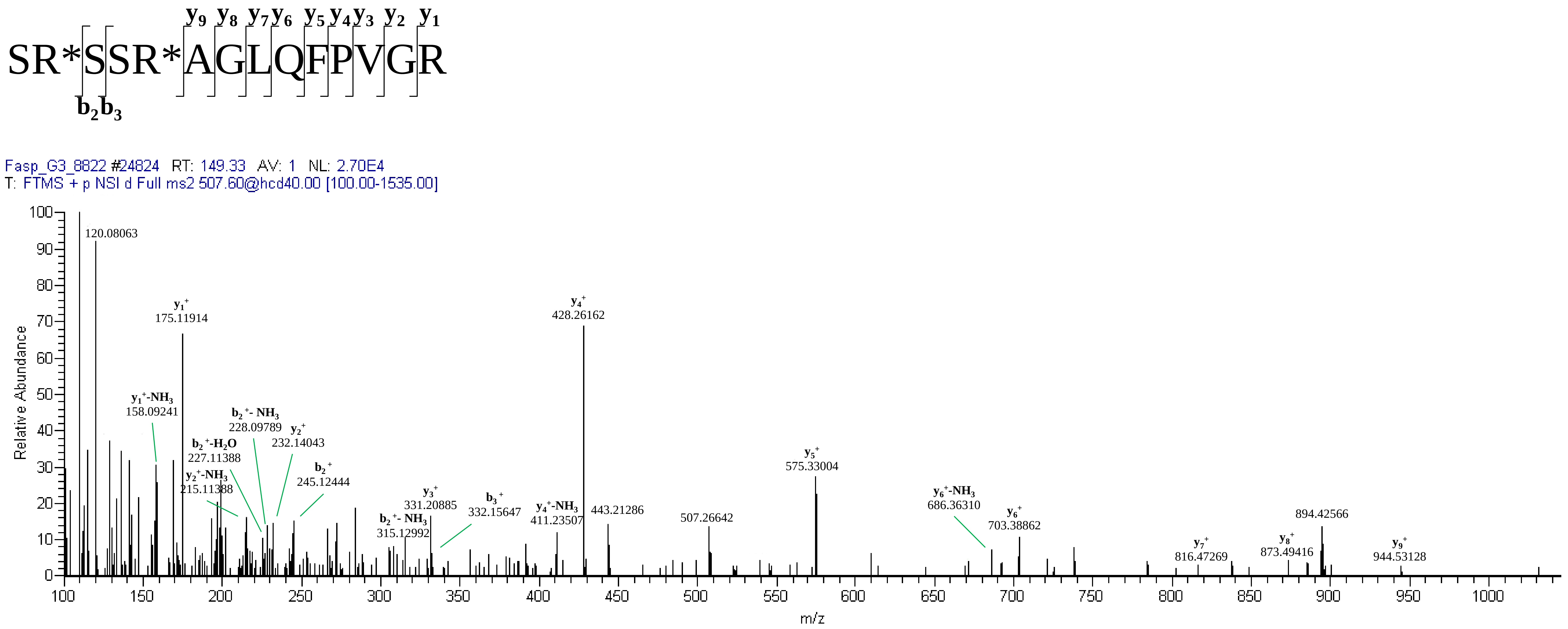

Mastitic B Replicate II

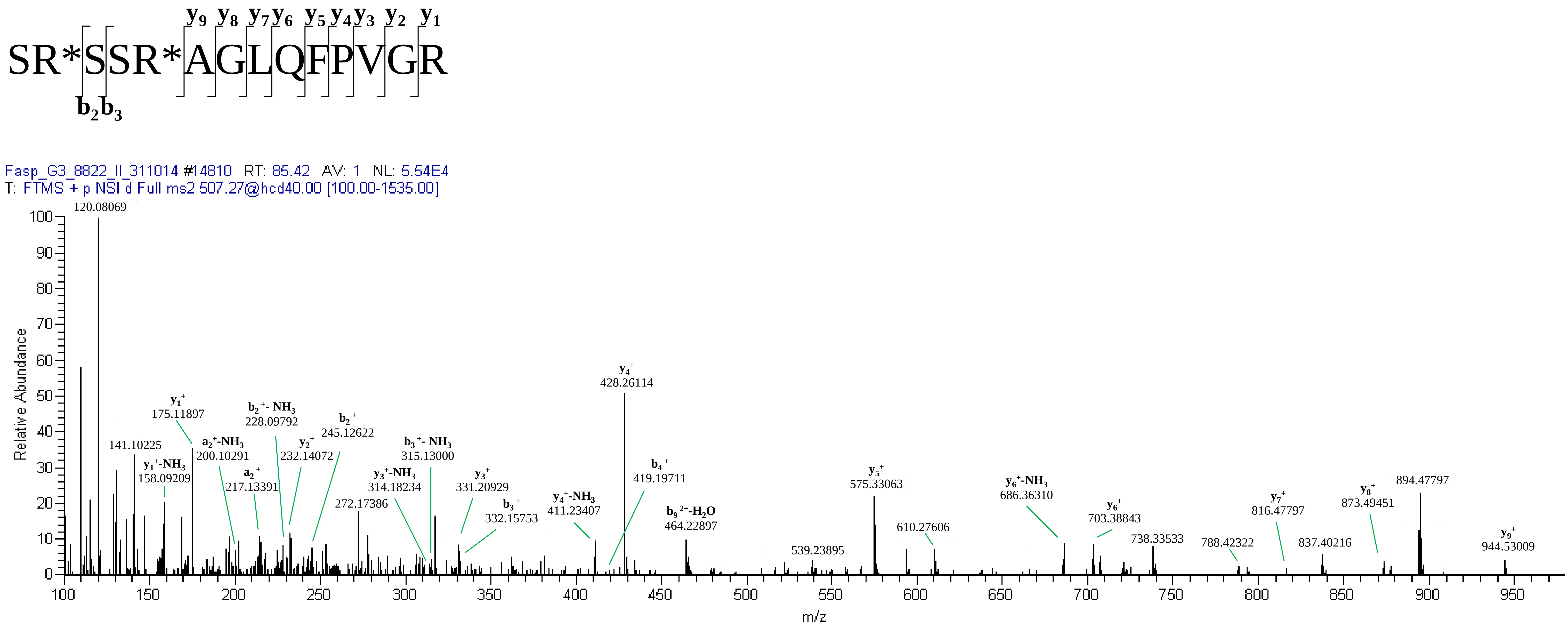

Mastitic B_Replicate I

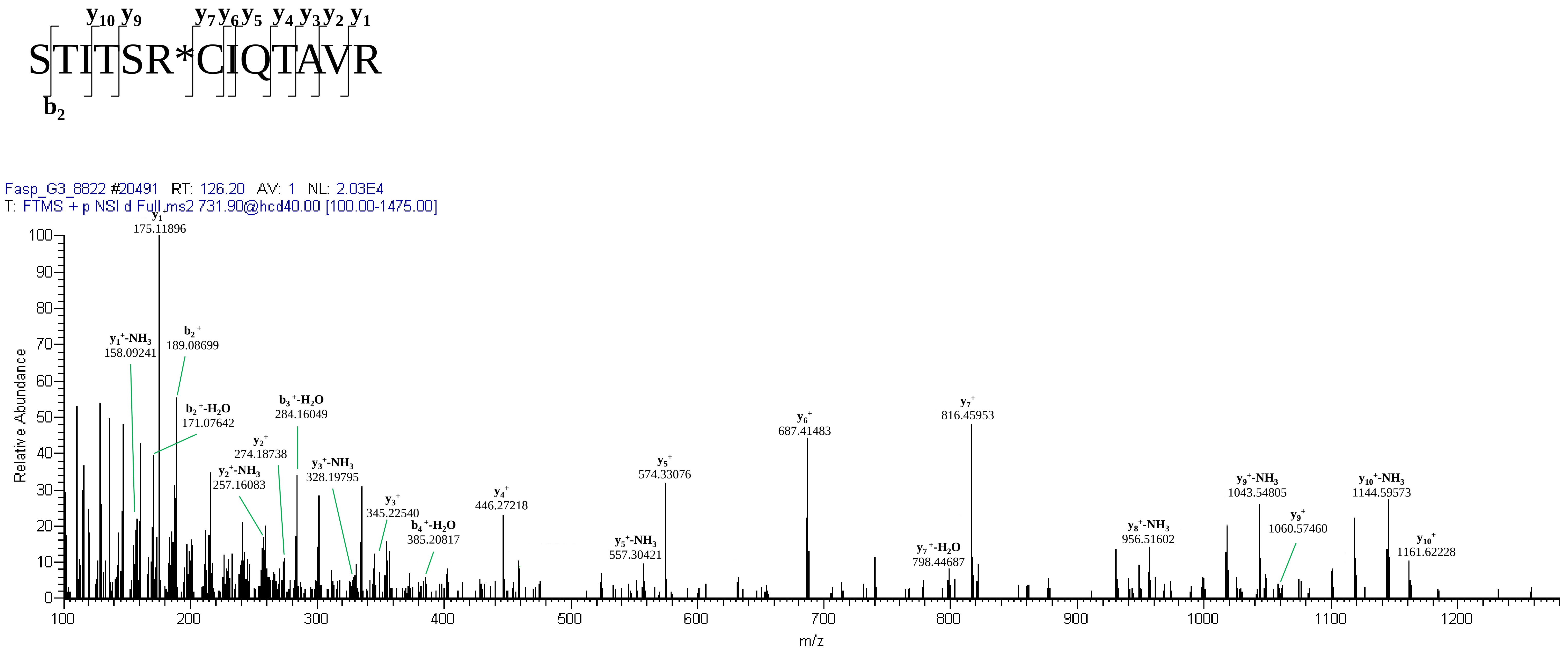

Mastitic B_Replicate I

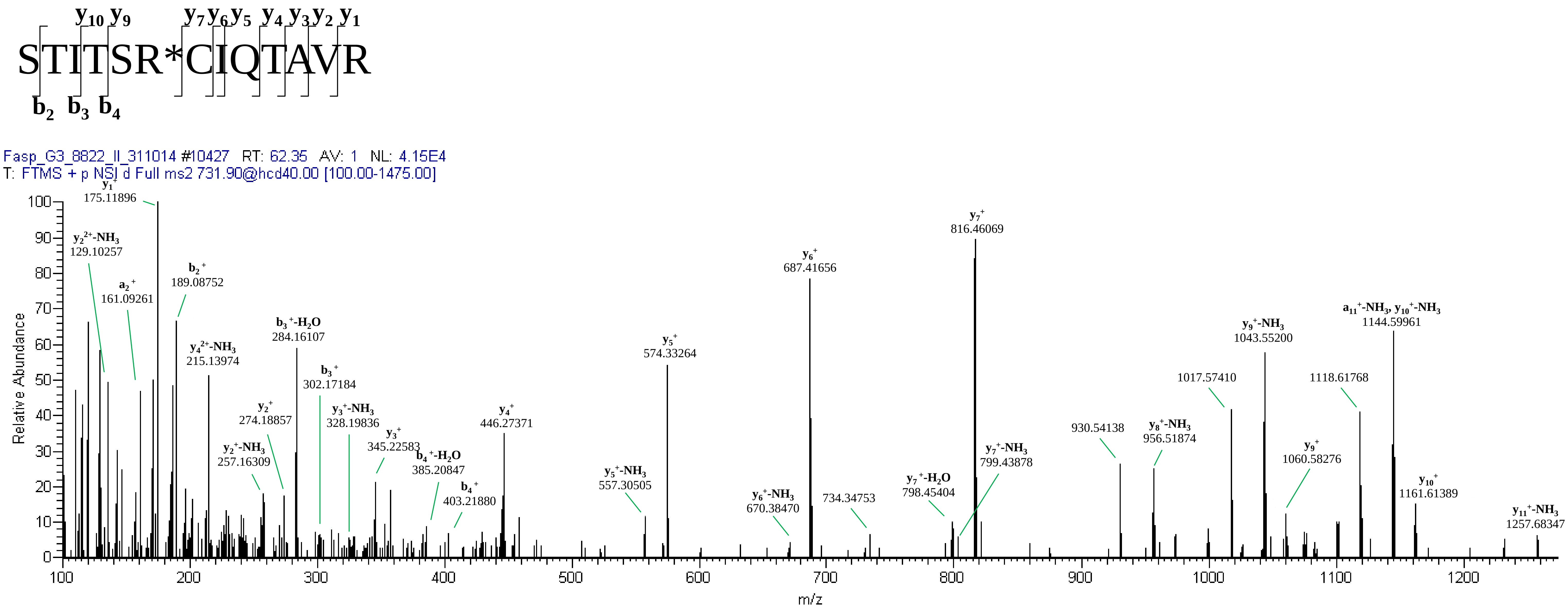

Supplement: Additional file 5: — Citrullinated peptide spectra. The spectra for citrullinated peptides detected in all samples are reported, together with detailed annotations. [file 13567_2015_196_MOESM5_ESM.pdf]
